# Aphasia recovery by language training using a brain–computer interface: a proof-of-concept study

**DOI:** 10.1093/braincomms/fcac008

**Published:** 2022-02-08

**Authors:** Mariacristina Musso, David Hübner, Sarah Schwarzkopf, Maria Bernodusson, Pierre LeVan, Cornelius Weiller, Michael Tangermann

**Affiliations:** 1 Department of Neurology and Neurophysiology, Medical Center—University of Freiburg, Faculty of Medicine, University of Freiburg, Germany; 2 Cluster of Excellence, BrainLinks-BrainTools, University of Freiburg, Germany; 3 Brain State Decoding Lab, Department of Computer Science, Technical Faculty, University of Freiburg, Germany; 4 Department of Radiology—Medical Physics, Medical Center—University of Freiburg, Faculty of Medicine, University of Freiburg, Germany; 5 Department of Radiology, Cumming School of Medicine, University of Calgary, Canada; 6 Department of Paediatrics, Cumming School of Medicine, University of Calgary, Canada; 7 Hotchkiss Brain Institute and Alberta Children’s Hospital Research Institute, University of Calgary, Canada; 8 Department of Artificial Intelligence, Donders Institute, Radboud University, Nijmegen, The Netherlands

**Keywords:** language training, chronic stroke, aphasia rehabilitation, neurofeedback training, brain–computer interface

## Abstract

Aphasia, the impairment to understand or produce language, is a frequent disorder after stroke with devastating effects. Conventional speech and language therapy include each formal intervention for improving language and communication abilities. In the chronic stage after stroke, it is effective compared with no treatment, but its effect size is small. We present a new language training approach for the rehabilitation of patients with aphasia based on a brain–computer interface system. The approach exploits its capacity to provide feedback time-locked to a brain state. Thus, it implements the idea that reinforcing an appropriate language processing strategy may induce beneficial brain plasticity. In our approach, patients perform a simple auditory target word detection task whilst their EEG was recorded. The constant decoding of these signals by machine learning models generates an individual and immediate brain-state-dependent feedback. It indicates to patients how well they accomplish the task during a training session, even if they are unable to speak. Results obtained from a proof-of-concept study with 10 stroke patients with mild to severe chronic aphasia (age range: 38–76 years) are remarkable. First, we found that the high-intensity training (30 h, 4 days per week) was feasible, despite a high-word presentation speed and unfavourable stroke-induced EEG signal characteristics. Second, the training induced a sustained recovery of aphasia, which generalized to multiple language aspects beyond the trained task. Specifically, all tested language assessments (Aachen Aphasia Test, Snodgrass & Vanderwart, Communicative Activity Log) showed significant medium to large improvements between pre- and post-training, with a standardized mean difference of 0.63 obtained for the Aachen Aphasia Test, and five patients categorized as non-aphasic at post-training assessment. Third, our data show that these language improvements were accompanied neither by significant changes in attention skills nor non-linguistic skills. Investigating possible modes of action of this brain–computer interface-based language training, neuroimaging data (EEG and resting-state functional MRI) indicates a training-induced faster word processing, a strengthened language network and a rebalancing between the language- and default mode networks.

## Teaser

The authors report on a novel language training for chronic stroke patients with aphasia. It utilizes a closed-loop EEG-based auditory brain–computer interface to reinforce suitable processing of word stimuli. A proof-of-concept study resulted in a medium to large and generalized language improvement independent of aphasia severity and speaking ability.

## Graphical Abstract

**Graphical Abstract:**
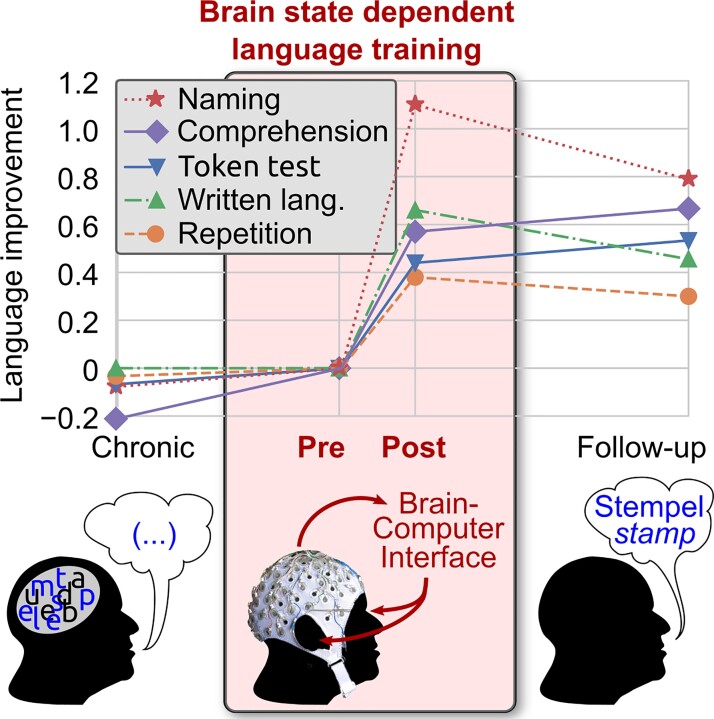

## Introduction

About one-third of stroke patients suffer from aphasia,^1^ a language impairment associated with a reduction or even loss of independence, social isolation^2^ and failure in returning to work.^3^

Spontaneous recovery of aphasia is observed within ∼6 months post-stroke, but only minimal improvements thereafter.^4^ In this chronic phase, conventional speech and language therapy (cSLT) achieves further recovery. In the most recent Cochrane meta-analyses, Brady *et al*.^5^ include 27 randomized studies that compare cSLT—including every formal intervention for improving language and communication abilities—versus no therapy in stroke patients with aphasia. The results showed clear evidence in favour of cSLT. However, the effects were short term only, with moderate effect sizes for functional communication, writing, reading comprehension, low ones for expressive skills and no effect for auditory comprehension and naming.^5^ Whilst therapeutic effects are similar by directly comparing diverse types of conventional therapies,^5,6^ stronger benefits for high-intensive cSLT are documented compared with low-intensive (<5 h/week) cSLT.^7^ Therefore, although there is no definitive agreement on the optimum amount, frequency or duration of treatment, intensive treatment is recommended^7–9^ and should be provided as intensely as possible whilst tolerated by the patient.^10^ Regarding more novel methodological approaches, there is currently no evidence of the effectiveness of the combination of cSLT and direct current stimulation (tDCS)—except for M1 tDCS^11^—versus sham tDCS.^12^ Despite all cSLT variants, ∼20% of stroke patients remain with persistent, communicative impairments.^13^

The combination of modest therapy success, severely reduced quality of life, a high-incidence rate (180 000 per year in the USA)^14^ calls for improved interventions.

In this study, we developed a novel therapeutic approach for aphasia rehabilitation using an EEG-based brain–computer interface (BCI). It utilizes machine learning to decode individual brain states in a single trial based on task-informative features, e.g. evoked potentials. Whilst BCIs traditionally decode intentions to substitute motor function, recent research focuses on motor recovery. Here, the idea is to provide immediate sensory feedback upon detecting an ‘appropriate’ brain state to reinforce re-learning strategies of the damaged brain using, e.g. functional electrical stimulation^15^ or robotic/orthotic devices.^16^ Such closed-loop BCI training protocols aim to reinforce the interaction between efferent and afferent brain networks. The feedback is causal and time-locked to the movement intent/attempt and not mediated by an external therapist. The results are promising: a meta-analysis for BCI-supported upper limb rehabilitation found a medium to large effect size^17^ and a recent randomized controlled trial has provided compelling evidence that the brain-state-dependent feedback is a key for the rehabilitation effect.^15^

Able to modulate networks, BCI feedback might even impact the language network. During cSLT, aphasic patients generally profit from feedback provided by the therapist or a computer programme. This feedback comprises confirmations of the adequacy of a patient’s response or providing corrective information regarding inadequate responses.^18^ Feedback helps the patient to improve and these improvements are associated with a functional reorganization of the language-related brain resource.^19,20^ A BCI approach does not require overt language production. It can provide feedback based on language-related brain activity. If this feedback also elicits therapeutic effects, it may open the door also for the treatment of other cognitive deficits that involve large variable networks.

Despite the current enthusiasm for BCIs, no BCI-based language therapy was reported. First, it was vastly unclear if auditory BCIs^21–23^ are feasible at all for patients with aphasia due to the rapid presentation of stimuli^24^ and their stroke-altered event-related potential (ERP) signals.^25,26^ Second, decoding intended speech from electrophysiological recordings—the analogy to BCI-based motor rehabilitation—is deemed a hard problem, although this topic has received growing attention.^27^ Third, the success of BCI (mainly) depends on reliable markers underlying the experimental task. Two ERP components of the EEG are highly related to specific aspects of language: the N400 component reflects comprehension,^26^ whilst the P600 component (reflects) syntactic processing.^28^ However, a novel therapeutic approach should be feasible and effective for most patients with aphasia, i.e. its beneficial effect should generalize to multiple aspects of language.

Thus, we opted for an easy task that requires a patient to process language to generate discriminative ERPs. We decided on a modification of the oddball task: patients had to listen to a cueing sentence whose last word was missing and then had to recognize the missing word (target) within a rapidly played audio sequence of several irrelevant (non-target) words. Existing literature proposed that detecting target versus non-target tones, phonemes or words is possible also in patients with severe language production deficits^29^ and can elicit discriminative ERPs, even if their characteristics deviate from that of controls. Moreover, amongst different ERP tasks, the oddball task was suited best to monitor recovery following global aphasia.

Most importantly, our word task is fundamentally different from classical tone-based oddball tasks. First, target words do not stand out by physical properties like pitch. They consisted of frequent concrete bisyllabic nouns with complex phonological, articulatory construction. Whilst listening to the rapid word sequences, patients should involve phonological and semantic competences to identify and differentiate target from non-target words. In line with this postulation, patients with low comprehension generally lacked such discriminative ERPs (mainly P300),^30^ confirming their association with semantic integration processing. Moreover, Cocquyt *et al.*^31^ documented a ‘phonological P300’ evoked by an oddball task with phonemes differing by their place of articulation. Second, our task required word recognition. Speech recognition literature proposes that higher-level lexical information activated by bottom-up phonetic input could be used to pre-activate upcoming potential phonemes before new bottom-up acoustic information arrives.^32^ Thus, we presumed that our task might activate neuro-motor programmes to control the articulators’ movements, i.e. the ‘internal speech’ in the Levelt model.^32,33^ Moreover, speech recognition can profit from (silent) repetition, which in turn involves aspects of speech production, including articulatory planning and execution.^34^ In addition, we know from neurocognitive studies^33–36^ and neuroimaging data^37,38^ that speech perception and internal speech (production) highly interact. Therefore, activation and positive reinforcement of word identification and recognition networks should also interfere with the speech production network. Third, performing the target detection in the BCI task surely demands general cognitive abilities such as attention, focused attention and executive functions possibly to a larger extent than traditional EEG experiments do,^39^ because of the concise word stimuli and the shorter stimulus onset asynchrony (SOA). These abilities are documented to be concomitant in patients with aphasia.^40^ However, it is still hotly debated whether the association of these deficits with aphasia is to the extent that they are part of the language system^40^ or that their anatomy largely overlaps with that of the language domain.^41^ Currently, language models and DTI data provide evidence that the language network is mainly localized in left-dominant fronto-temporo-parietal (FTP) brain regions connected by the dorsal and ventral pathways.^42–46^ Within this model, it has been proposed that focused attention- or intention-related changes in the inferior parietal lobe influence the selection of context-dependent action programmes in the prefrontal and premotor cortex.^46–48^ It has been shown that a left hemispheric stroke can cause aphasia directly by damaging the dual language system,^49^ but it also can cause a disbalance between the default mode network (DMN) and the language FTP network.^50^ In the healthy population, the DMN is considered a domain-general system for cognitive control and attention. As a task-negative network,^51^ the DMN shows reduced activity during task performance, e.g. upon language comprehension or production. A stroke-induced upregulation of the intact DMN and downregulation in the lesioned FTP are partially reversed again upon the improvement of the speech production.^50^

All these scientific data led us to conclude that our training task is definitively word-related and involves computations of bottom-up encoding, top-down control for matching the input with an internal linguistic expectation context, and indirectly, propositional speech. However, we were not sure to what extent our task reflects general auditory processing and cognitive abilities as those involved in pitch recognition and counting during the classical oddball task. Therefore, we included not only a classical tone-based oddball task but also tested cognitive functions. Even though the main goal of our study was not to distinguish the different mechanisms of aphasia’s recovery, we found the hypothesis of the imbalance between different brain networks relevant for further investigations. Thus, we decided to also include a repeated resting-state functional MRI (rs-fMRI) measurement.

During the BCI-based training task, an individually trained classifier analyses the ongoing EEG. To infer the attended word stimulus, the classifier exploits any systematic differences between target and non-target word ERPs. Based on how well the classifier could identify the attended word and the accordance between attended and target word, the BCI presents feedback that informs the patient if the currently applied processing strategy generates ERP differences (see the ‘Materials and methods’ section). This feedback may provide patients with indirect information about the activation of the patient-specific brain networks contributing to solving the language task. The reinforcement of these networks may be beneficial, but they are not deliberately accessible by patients or therapists and thus cannot be utilized by existing forms of language training. Note that the BCI positively reinforces word-evoked ERPs only, whilst patients do receive feedback neither during nor after the classical tone-based oddball task. Several studies found that, although the discriminative ERP components were similar within the diverse oddball tasks, dissociation of brain ERP topographies is well documented, for example, for tonal and phonetic tasks.^52,53^ Thus, we expected that our protocol might selectively train up those language skills—basically sensorimotor language competence—underlying our task, instead of general executive function.

We implemented this BCI approach in a training protocol consisting of 4 training days a week for an overall amount of 30 h of effective training, with 10 chronic stroke patients with different aphasia severities and types, to investigate the following hypotheses: (H1) Our fast word-based BCI task is feasible not only with healthy participants but also with patients having aphasia, and patients’ single-trial word ERPs are sufficiently informative to provide feedback. (H2) An intensive training using this BCI-based feedback can improve language. (H3) Obtained improvements will be language-specific.

Additional 20 controls underwent a single session to investigate the feasibility and discriminability of patient ERP data (H1). Effectiveness (H2) concerning multiple language competences including functional communication was analysed at multiple time points by standardized language tests. Data on cognitive abilities, ERP responses and functional connectivity (FC) based on rs-fMRI were evaluated to investigate the training’s specificity (H3) and to gain an understanding of its effects.

## Materials and methods

### Patients and controls

**Figure 1:**
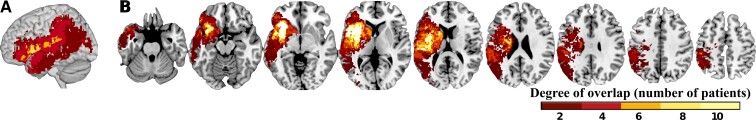
**Overlap of the binarized lesions of the 10 patients**. Lesions are displayed over sagittal surface (**A**) and over nine horizontal sections parallel to the AC-PC line (**B**). Brighter regions indicate a greater degree of overlap of lesions. Images were generated using mricron.

Ten patients (age 58 ± 11 years, one female) with different types of chronic aphasia caused by a single left hemispheric ischaemic stroke (see Fig. 1) were recruited at the University Medical Center Freiburg and 20 right-handed normally aged controls (NACs, age 60.2 ± 8 years, 10 females). Both studies were approved by the ethics committee of the University Medical Center Freiburg. All subjects gave written informed consent in agreement with the Declaration of Helsinki. The patient study was registered in the German Clinical Trials Register (DRKS00013572). All participants were right-handed native German speakers without prior experience with auditory BCI paradigms. For patients, the inclusion criteria were: (i) single left hemispheric ischaemic stroke affecting the left middle cerebral artery; (ii) time point of stroke at least 6 months prior to start of training; (iii) presence of aphasia confirmed by Aachen Aphasia Test (AAT); (iv) age 18–80 years and (v) sufficient cognitive abilities to understand and work with the experimenters and provide informed consent. Exclusion criteria were: (i) a bilateral stroke; (ii) haemorrhagic stroke; (iii) additional structural brain lesions (tumour, trauma, high cerebral microangiopathy) detected in the MRI; (iv) high cerebral artery stenosis; (v) history or current diagnoses of other medical, neurological or psychiatric disorders interfering with participation or data analysis; (vi) severe hearing deficit; (vii) vision loss; (viii) factors hindering fMRI or EEG acquisition; (ix) early bilinguals; (x) professional musicians and (xi) left handedness. Language understanding deficits were not considered an exclusion criterion, if (after familiarization) the training task could be executed. NACs were required to have no known history of stroke or other neurological or psychiatric disorders. Moreover, we applied the same exclusion criteria as for patients. We did not aim to compare both groups. Patients were recruited to investigate the effect of training, whilst NACs served to obtain example ERP responses for the newly designed word-based paradigm. Note that sex was imbalanced in the patient group and that ERP characteristics may vary depending on sex^54^ and other factors. For our training approach, however, the presence of discriminative ERPs alone was relevant, whilst inter-individual or any group-dependent ERP differences were not relevant, as the individual machine learning model was trained.

A full overview of the behavioural and demographic patient characteristics is given in Table 1. Patients showed concomitant focal neurological symptoms and most patients showed cognitive deficits, see Supplementary Table 1.

**Table 1:** Overview of patient-specific information to demography, stroke, aphasia and comorbidities

Demography				Stroke-related information									Aphasia-related information (before training)			Others
	Sex	Age	Edu. age	Stroke aetiology	Stroke risk factors	Acute therapy	Stroke severity (mRS) at T0, T1, T2	Infarct volume (ml)	MCA stroke location	Additional stroke location	Months post-stroke at training start	Hemi- paresis (severity)	Aphasia severity	AAT-based aphasia type	Speech apraxia severity	Comorbidity
1	m	76	11	CE, LAA	H, AF, D	ly, mr, s	3/2/2	113	F, T, P, In		10		Medium	Broca		
2	m	58	17	LAA	H		4/4/2	13	F, P, In, NC	ACA, AChA	18	Severe	Mild	Anomic	Mild	
3	m	71	23	LAA	H, CHD	s	4/3/2	43	F, T, P	ACA	36	Mild	Mild	Anomic		Epilepsy, MM
4	m	70	11	EO	H		4/4/3	47	F, T, P, In		9	Severe	Mild	Broca		Prostate cancer
5	m	60	12	LAA	H, HL, N	ly, mr, s	5/4/2	68	F, T, P, In, NC		27		Mild	Anomic		
6	m	43	19	LAA	H, HL	ly	3/3/2	125	F, T, P, In, NC	PBZ	10	Severe	Mild	Broca	Medium	Epilepsy
7	f	54	23	ICA-D		ly, mr, s	5/4/2	100	F, T, In, NC	ACA	8	Mild	Mild	Anomic		Depression
8	m	61	17	ICA-D	H, HL		3/2/1	87	FT, In		149		Mild	Anomic		
9	m	38	12	CE		he	5/3/3	217	F, T, P, In		21		Severe	Broca	Mild	
10	m	53	12	CE	H, HL	ly, mr, he	5/5/3	145	F, T, P, In		12	Severe	Severe	Global	Mild	Depression

AAT refers to the Aachen Aphasia Test.

Edu. age refers to the educational age, i.e. the number of years in school and in higher education.

Stroke aetiology of (ischaemic) stroke subtype: cardioembolism (CE), large-artery atherosclerosis (LAA), internal carotid artery dissection (ICA-D) and embolic undetermined aetiology (EO).

Risk factors: atrial fibrillation (AF), coronary heart disease (CHD), diabetes (D), hypertension (H), hyperlipidaemia (HL), nicotine (N).

Stroke severity was assessed with the modified Rankin Scale (mRS) at stroke admission (T0)/discharge (T1)/before training (T2).

Acute therapy consisted of thrombolysis (ly), mechanic recanalization (mr), ACI Stent (s) or hemicraniectomy (he).

Location of stroke: all patients exclusively had a single stroke in the middle cerebral artery territory. Within its reach, strokes affecting frontal (F), temporal (T), insula (In), parietal (P) and nucleus caudatus/thalamus (NC) regions are distinguished. In some patients, the same embolic stroke also affected the anterior cerebral artery (ACA), anterior choroidal artery (AChA) or posterior border zone (PBZ).

AAT-subtype. Anomic: mild form of aphasia with difficulties to name objects; Broca: partial loss of the ability to produce language (spoken and written); global: most severe form of aphasia heavily affecting comprehension and production.

Apraxia of speech refers to a disorder which affects an individual’s ability to translate conscious speech plans into motor plans.

Comorbidity: multiples myeloma (MM).

### Study design

**Figure 2:**
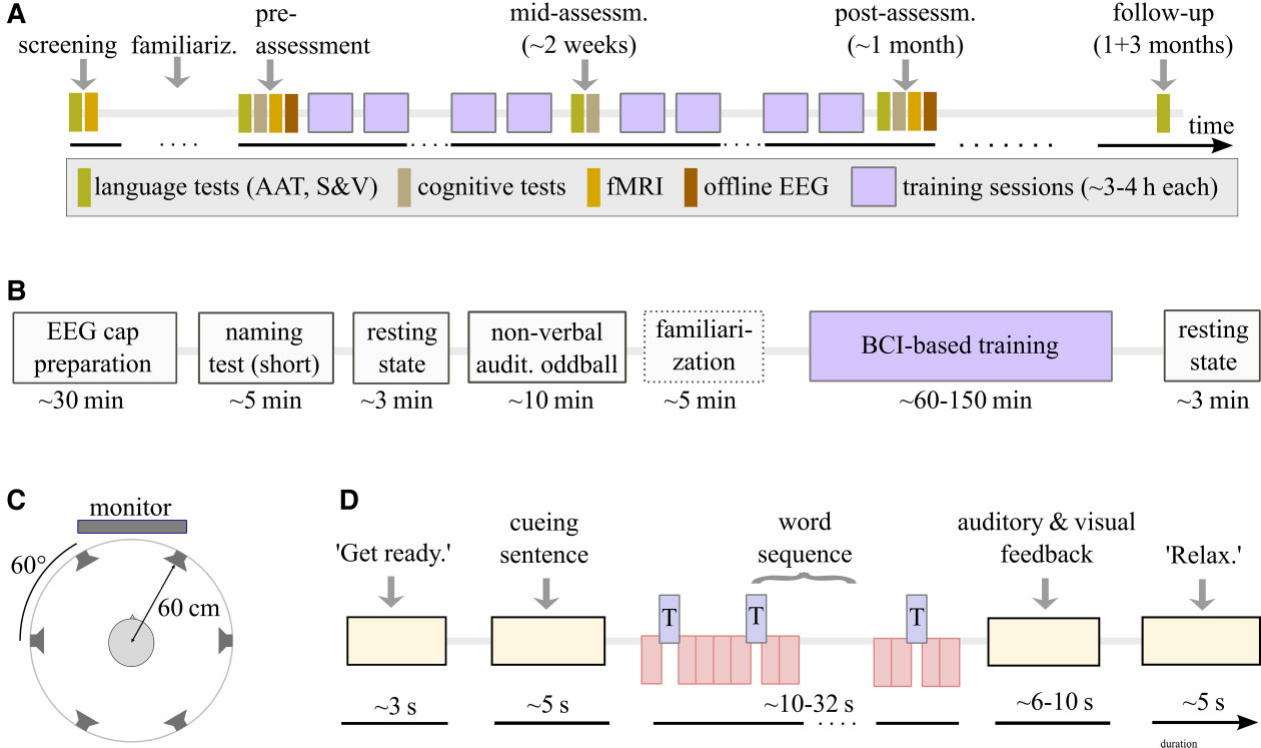
**Study protocol for BCI-based language training**. (**A**) Time points (relative to the first training session) of the clinical testings and training sessions that each patient underwent, including an individual familiarization with the paradigm (hours to few sessions per patient), language assessments (AAT, Aachen Aphasia Test; S&V, Snodgrass & Vanderwart naming test; CAL, communicative activity log) and cognitive assessment (TAP, test of attentional performance; digit span, Corsi span, semantic and phonological fluency), see Supplementary Section 2. (**B**) Structure of a single training session and duration of its components. (**C**) Set-up of the AMUSE protocol^23^: a subject is placed in the centre of six loudspeakers placed at ear level. In this loudspeaker condition, all auditory stimuli and auditory feedback were presented over these loudspeakers. Within each trial, a 1:1 relation was maintained between the six words and the loudspeakers. Between trials, the target word and the mapping between directions and loudspeakers were pseudo-randomized. In a headphone condition, the patient received sentences, word stimuli and auditory feedback in one mono-channel via headphones such that spatial information could not be exploited. (**D**) Structure of a single trial, consisting of a ‘get ready’ cue, a sentence presentation, a word sequence presentation, immediate EEG analysis and feedback to the patients. At trial start, the computer played one of six German cueing sentences from an audio file, but the sentence’s last word was missing. Example: ‘*Die neue Tonerkartusche steckt schon im … *’ (‘*The new cartridge is already in the *… ’). During the sentence presentation, patients were asked to listen to and understand it with the goal to infer the target word. Following, a sequence of six different words (all bisyllabic nouns with durations below 300 ms) was played. It took about 32 s (for SOA 350 ms if no dynamic stopping was triggered) and consisted of 15 target words (blue rectangles, Drucker/printer in this example) and 5 × 15 = 75 presentations of the five non-target words (red rectangles). The target and non-target role of a word switched in a pseudo-randomly balanced manner between trials of the same run.

Our study protocol (Fig. 2) aimed to monitor communication difficulties and to differentiate linguistic from non-linguistic skills. Following clinical guidelines for stroke-induced aphasia rehabilitation^8,9^ and comparability, we included assessments considered validated, reliable and frequently used in aphasia research. To assess language functions (at screening/pre/mid/post/follow-up), we used the AAT,^55^ consisting of six description levels for spontaneous speech and five subtests (token test, repetition, written language, naming and comprehension). This clinical tool is validated and standardized for the German language.^56,57^ It provides confidence intervals, percentile scores and *T*-score units for each subtest and has a good test–retest and inter-rater reliability.^58^ Thus, the AAT allows us to quantify significant individual improvements of specific linguistic skills and to monitor clinical severity of aphasia in an individual patient, according to Huber’s psychometric analysis. For detailed naming assessment, we included the widely used^59,60^ Snodgrass & Vanderwart (S&V) picture naming test^61^ (for details, see Supplementary Section 1). For AAT and naming tests, correct responses were never provided to patients. To capture deficits in everyday life situations, patients and relatives carried out the Communicative Aphasia Log (CAL), a questionnaire quantifying and qualitatively describing patients’ communication activities,^62^ before and after the training.

Patients underwent the following cognitive tests: the subtests alertness, attention and flexibility of the standardized ‘test of attentional performance’ battery,^63^ digit span,^64^ Corsi span^65^ and semantic and phonological fluency^66^ (Fig. 2A and Supplementary Section 2).

Before and after the training, rs-fMRI scans (6 min each) were acquired by a 3 T Magnetom Prisma scanner (Siemens Healthineers, Erlangen, Germany) using multiband-accelerated echo-planar imaging sequences (multiband factor 3), 220 mm × 220 mm field of view, 128 × 128 matrix size, 84 axial slices (1.7 mm thick), repetition time = 1.54 s, echo time = 27 ms, flip angle 79° and 240 volumes. In addition, anatomical images (T1 MP2RAGE, 1 mm resolution) and diffusion-weighted echo-planar imaging images (1.7 mm resolution, 61 diffusion directions, *b* = 0, 1000 s/mm²) were acquired for normalization and lesion segmentation purposes, respectively.

Three EEG sessions without feedback were conducted: two before the first training session to tune stimulation parameters and one after training completion. The online EEG training with feedback was delivered 4 days a week and as intensively as possible until 30 h (similar to other studies)^7,62,67^ of effective training time were reached.

Our study was not randomized and a cSLT was not part of our protocol. However, as usual in Germany, all patients except P8, who suffered from a mild anomic aphasia for more than 12 years, had received cSLT from local therapists at Freiburg and surrounding places in the time between screening and the start of the BCI-based training (Fig. 2). Typically, this cSLT followed a deficit-oriented training approach. Note that we did not influence how a patient’s therapist conducted the cSLT. The pre- versus post-comparisons of cSLT and AAT results, nevertheless, allowed us to describe the effect of the conventional therapy upon language skills.

During the 3 months after our training and prior to our follow-up, patients were not allowed to participate in any form of language or speech therapy.

### Structure of a training session

The time course of a patient’s training session is depicted in Fig. 2B. A multi-channel EEG was set up (see Supplementary Section 3 for details) before patients were asked to name around 25–30 pictures (results not reported). None of the items of this short test overlapped with items of the S&V or AAT. Patients underwent two non-verbal auditory oddball runs with a high or low tone played every second, each containing 50 high-pitched target tones and 250 low-pitched non-targets.

Subsequently, patients performed multiple runs of the auditory BCI-based training task, see Fig. 2C. It extended the AMUSE paradigm^23^ in three ways: first, we allowed for variations of the stimulus timing, expressed by SOA between 250 and 1000 ms. Second, a mono-presentation of stimuli via headphones (headphone condition) could be used alternatively to the spatial auditory presentation over six loudspeakers directions (loudspeaker condition) in AMUSE. Third, we used bisyllabic word stimuli in our study instead of short tones. The target and non-target words were selected pseudo-randomly from a pool of words. They all had the same duration and had been recorded by the same male native German speaker. The six words were difficult to articulate, as their first syllable contained a consonant cluster of which the consonants belonged to different articulatory classes, e.g. alveolar-plosive and uvular-fricative as in ‘Trichter’ (funnel). The consonant or consonant cluster of the second syllable again belonged to a different articulatory class or classes.

Depending on the SOA, a single trial of the training task took 35–60 s, see Fig. 2D. After a run of six trials, patients could take a break. The number of runs per session was adapted to the stamina of the patient.

During a trial, the patient was asked to focus on presentations of the target word whilst ignoring non-target words presented in a sequence. The target word was cued uniquely by a preceding sentence (see Supplementary Section 4). As overt word production or other behavioural responses were never required, only the BCI output could inform experimenters about whether the task was accomplished or not.

Using signal processing and machine learning methods (see the ‘EEG analysis’ section), the BCI classifier continuously analysed systematic target- versus non-target ERP differences to identify the word attended by the patient and to verify if the attended word was the correct target word for this sentence. More specifically, classifier outputs (i.e. distances to the decision hyperplane) were used as surrogates for how well a patient’s brain could discriminate between target and non-target words. Within one trial, outputs for up to 15 presentations were collected separately for each of the six words. At trial end, the patient received feedback on how clearly the classification outputs of targets and non-targets differed. In the case of accordance between identified word and target word, the patient received a positive, reinforcing auditory (sentence) and visual (screen visualizations) feedback (for details, see Supplementary Fig. 1). It informed the patient that the currently applied processing strategy was a successful one. Detecting insufficient differences in ERPs between target and non-target words or when the detected word did not correspond to the target word, patients received neutral auditory and visual feedback informing him/her about the correct target.

After the session, a speech therapist, neurologist, or other academic staff could support patients to recognize potentially successful individual strategies to solve the task.

Each NAC performed a single offline EEG session without feedback, without dynamic stopping, with SOA = 250 ms and at constant difficulty. For details, see Supplementary Section 5.

### Adapting task difficulty over time

For patients, throughout a session, either the loudspeaker or the more challenging headphone condition was applied, and a constant SOA and the same six sentences and six words formed the stimuli. Between sessions, the task difficulty could be adapted by modifying multiple experimental parameters (see Supplementary Section 6), to maintain a high task demand.

### Behavioural analysis

AAT performances pre- versus post-training and versus follow-up were assessed to investigate training-induced effects. The comparison of AAT performances at screening versus pre-training time points was performed to investigate the effect of preceding cSLT. We observed a stable clinical profile of aphasia in all 10 chronic patients. S&V scores comprised correctly named words, latencies, phonetic and semantic five-point scores (see Supplementary Section 1 for a detailed description of the evaluation). Pre-training scores were compared against mid-, post-training and follow-up. For the other behavioural tests listed in the study design section, we compared only pre- versus post-training performances.

Based on their relevance for our training task, five of the conducted cognitive tests were statistically evaluated: (i) the digit span test,^64^ assessing working memory; (ii and iii) the visual Go/NoGo-task’s reaction time and the number of errors, testing selective attention and (iv and v) the median reaction time to a visual stimulus with and without a prior auditory warning signal, measuring alertness. See Supplementary Section 2 for details.

### EEG analysis

The processing of EEG data (63/31 channels for offline/online sessions, respectively) and the training and evaluation of classification models were conducted in MATLAB version 2014b using the BBCI toolbox^68^ with a standard pipeline established for ERP analysis (see Supplementary Section 3).

To classify target from non-target ERP responses during the patient training, one individual regularized linear discriminant analysis (LDA) model^69^ was maintained per patient. Using transfer learning, this model was pre-trained on data of the two offline sessions for the first online session. Therefore, any previous online session provided pre-training data for the upcoming next online session. Within sessions, the model was repeatedly updated after each run by supervised adaptation^70^—the latter was possible as the true labels were known at any time. The adaptation was supposed to account for non-stationary feature distributions over time, which could be caused by patient learning or fluctuations of the vigilance. The adaptation parameters *η*_1 _= 0*.*005 (for the class mean estimation) and *η*_2 _= 0*.*001 (for the global covariance matrix) had been determined from the NACs’ offline data.

Maximizing the reinforcing feedback per session, we allowed for stopping a trial’s word sequence even prior to the presentation of full 90 words and a very positive feedback could be triggered in these cases. For details of this dynamic stopping strategy, see Supplementary Section 3 and Fig. 1.

For any offline classification analysis reported, individual LDA models were trained per patient. We performed a 5-fold chronological cross-validation to assess a model’s accuracy, which is expressed as the area under the receiver-operating characteristic curve (AUC). A value of 0.5 [range: (0, 1)] indicates chance level performance and 1.0 perfect classification. ERP peak amplitudes and latencies were estimated in a bootstrap procedure where 80% of the data was randomly sampled 10 times and peak readouts were then averaged. The P300 onset was determined as the first time point when the target and non-target responses differed significantly based on a two-sided *t*-test (*α *= 0*.*05) and set to 1000 ms if no such difference could be observed. Non-verbal auditory oddball ERPs were analysed similar to word ERPs.

### Functional MRI analysis

Patients’ rs-fMRI data were motion-corrected, normalized to MNI space and smoothed with a 6 mm Gaussian kernel using the FSL toolbox.^71^ The normalization step was performed by first co-registering the functional images to the anatomical images with a rigid-body linear transformation and then normalizing the anatomical images to 2 mm MNI space with an affine linear transformation, with mutual information as the cost function. Visual inspection of all transformed images confirmed the accuracy of the registrations in spite of the sometimes extended lesions in some patients, so that further adjustments were not necessary. Functional time series were then extracted from multiple seed region of interest (ROI) from the default mode and the language network after regressing out the average white matter and cerebrospinal fluid time courses and bandpass filtering between 0.01 and 0.1 Hz. Only non-lesioned voxels within each ROI were included in the calculation of the fMRI time series. The lesions had been manually segmented on the apparent diffusion coefficient maps derived from the diffusion images. All considered ROIs included at least 82 non-lesioned voxels, so that ROI time series could be reliably extracted in all cases. The ROIs included the posterior cingulate cortex, precuneus, F3op, F3tri and F3orb from the AAL atlas^72^ and the posterior superior temporal gyrus from the FIND atlas.^73^ FC was defined as the Fisher-transformed correlation coefficient between the seed ROI time courses and those of every (non-excluded) voxel in the brain. Differences between pre- and post-training FC were assessed by a paired *t*-test with a voxel-wise significance level of *α* = 0*.*001 using an additional cluster-extent threshold corresponding to a family-wise error rate of *α *= 0*.*05. Laterality indices were computed on the statistical maps as described in Supplementary Section 7.

### Statistical analyses

Statistical analyses were conducted in R v3.3.1 (standard package) and Python v3.6 using the libraries NumPy (v1.15.4), pandas (v0.23.4) and SciPy (v1.2.1). To assess statistical significance, we used two-sided *t*-tests for normally distributed quantities and non-parametric tests otherwise. Paired tests were applied when suited. The specific choices of the statistical tests are detailed in the ‘Results’ section. To control the false discovery rate in multiple comparisons, we applied a Benjamini–Hochberg correction. To limit the number of comparisons, the testing of pre-versus post-training cognitive performances was restricted to the above-mentioned five cognitive tests (see ‘Behavioural analysis’ section).

Following Cochrane recommendations,^5^ we calculated all effect sizes as Hedges’ *g*_s_ (see Supplementary Section 8). As T-transformed AAT scores are designed to have a standard deviation of 10, we directly used that value instead of estimating the population standard deviation, resulting in an effect size given by

$$d_{s}\;:=\frac{\mathrm{Mean}(z_{\mathrm{post}})-\mathrm{Mean}(z_{\mathrm{pre}})}{10}$$

with ***z***_pre_ and ***z***_post_ denoting the pre- and post-assessment values. We empirically confirmed that the estimated standard deviation is close to 10, see Table 2. For all other quantities, we estimated the population standard deviation.

**Table 2:** Training-induced effects are language-specific and generalize beyond the training task

Category and tests	*N*	Pre-training	Post-training	Raw *P*-value	Effect size
**Aachen Aphasia Test**		**Mean (SD)**	**Mean (SD)**	**Paired *t*-test**	***g*_s_**
Token test (*T*-score)	10	58.90 (13.25)	63.30 (11.40)	**0.0023**	0.44
Repetition (*T*-score)	10	56.90 (8.33)	60.70 (9.31)	**0.023**	0.38
Written language (*T*-score)	10	55.40 (7.46)	62.00 (10.87)	**0.0033**	0.66
Naming test (*T*-score)	10	56.50 (8.28)	67.50 (13.48)	**0.0019**	1.1
Comprehension (*T*-score)	10	59.10 (8.13)	64.80 (13.26)	**0.0232**	0.57
		**Median (range)**	**Median (range)**	**Signed-rank test**	**Hedges *g*_s_**
Sum of spontaneous speech subtests: (0–30)	10	24 (14–29)	26 (16–30)	**0.0072**	0.52
**Naming abilities (S&V naming test)**		**Median (range)**	**Median (range)**	**Signed-rank test**	**Hedges *g*_s_**
Correct words (%)	10	59 (9–70)	65 (16–76)	**0.0039**	0.28
Semantic score (0–4)	10	3 (2–4)	3 (2–4)	**0.0098**	0.21
Phonological score (0–4)	10	3 (1–4)	4 (2–4)	0.0371	0.16
Semantic access delay (s)	10	1.7 (1.1–2.3)	1.5 (0.89–3.0)	0.375	0.2
Phonological access delay (s)	10	1.6 (1.1–2.5)	1.7 (.89–3.6)	0.4316	0.33
**Functional communication (CAL)**		**Mean (SD)**	**Mean (SD)**	**Paired *t*-test**	**Hedges *g*_s_**
Quantitative (sum)	10	28.90 (11.10)	34.30 (11.45)	**0.0003**	0.46
Qualitative (sum)	10	76.90 (25.45)	84.40 (24.48)	**0.0002**	0.29
**Cognitive tests**		**Median (range)**	**Median (range)**	**Signed-rank test**	**Hedges *g*_s_**
Digit span (total count)	9	8 (0–12)	9 (0–13)	0.8867	–0.03
Go/NoGo (number of errors)	10	4 (1–31)	4 (0–36)	0.726	–0.01
Go/NoGo (ms)	9	548 (417–944)	596 (461–700)	0.7344	–0.06
Alertness without signal (ms)	10	248 (201–358)	282 (218–483)	0.0371	0.59
Alertness with signal (ms)	10	263 (188–374)	287 (218–366)	0.5071	0.27

Following common practice, raw AAT scores were initially transformed into normally distributed *T*-scores with a mean of 50 and a standard deviation of 10. For the *T*-scores and other metrics, we report the mean and standard deviation (SD) for approximately normally distributed quantities and median and range otherwise. Reported *P*-values are not corrected for multiple testing. Bold *P*-values indicate significant changes *after* correcting for multiple comparisons with the Benjamini–Hochberg correction at an *α*-level of 0.05. We corrected for multiple tests within each category, i.e. for six tests in AAT, five in S&V naming, two in functional communication and five in cognitive tests. Effect sizes are calculated as the mean difference divided by the population standard deviation (which is taken as 10 for the *T*-scores to obtain *d_s_* and estimated for all other quantities to obtain Hedges *g*_s_, see the ‘Materials and methods’ section). AAT, Aachen Aphasia Test; S&V, Snodgrass & Vanderwart naming test; CAL, communicative activity log; signed-rank test, Wilcoxon signed-rank test.

This is a one-armed proof-of-concept study. Thus, neither randomization nor blinding was performed, and power analysis had not been performed. The priority of our paper was to investigate training-induced effects within the group of patients. Therefore, we restricted any statistical comparisons to pre- versus post-effects observed within the patient group, which also reduced the correction factors for multiple testing.

### Data availability

The data supporting the findings of this study are available from the corresponding author upon reasonable request.

## Results

### Feasibility

All screened patients fulfilling the criteria were included. No included patient dropped out, but we decided to stop patient P2’s training after 24 h due to undesired finger motor activity (see Supplementary Section 9). A short familiarization of maximally 2–10 h for patients with severe aphasia was sufficient to prepare the training. Application of the EEG cap, the high training frequency and intensity and task monotony were not considered real problems. The effective feedback training duration (excluding cap preparation, assessments and resting state recordings, see Fig. 2) was 6.26 h on average per week and 14.9 sessions were required on average to reach 30 h (for details see Table 3). Patient P7 initially was exhausted quicker than the nine other patients and needed substantially more sessions, but rapidly learned to deliver longer sessions.

**Table 3:** Summary of training- and aphasia-specific patient data

Patient	cSLT before BCI training			BCI training		Total AAT points and severity of aphasia					
Number	Times per week (*45 min)	Duration		Number of sessions	Duration in effective hours	Chronic phase	Pre-training		Post-training		Follow-up
		Days	Effective hours			AAT points before cSLT	AAT points	Severity	AAT points	Severity	AAT points
1	3	103	25.4	15	35.3	321	318	Moderate	374	Moderate	374
2	2	356	58.5	11	24.2	490	495	Mild	517	No aphasia	x
3	x	x	x	14	30.0	x	471	Mild	523	No aphasia	518
4	2	48	7.9	17	29.7	470	457	Mild	503	No aphasia	475
5	3	57	14.1	11	29.3	448	467	Mild	519	No aphasia	508
6	3	423	104.3	13	30.0	430	448	Mild	494	Mild	473
7	3	64	15.8	25	30.2	446	468	Mild	492	No aphasia	504
8	0	300	0.0	15	29.5	473	466	Mild	498	Mild	503
9	4	48	15.8	15	30.4	243	245	Severe	276	Severe	291
10	3	117	28.8	13	30.0	181	198	Severe	240	Severe	254
Avg (SD)	2.6 (1.1)	168 (149)	30.1 (30.6)	14.9 (4.0)	29.9 (2.6)	389 (113)	403 (108)		444 (107)		433 (101)

From left to right: 9 out of 10 patients underwent cSLT before starting the BCI training. The AAT severity ratings before cSLT and at the pre-training time point were identical. We report on the intensity (times per week), duration (in days) and effective training hours of this cSLT between the assessment in the chronic phase and the start of the BCI training (pre-training assessment). Then, patients had 11–25 BCI training sessions. The average effective training time (accidentally) was the same for cSLT and BCI training. Note that cSLT differs from BCI training not only regarding the tasks but also regarding the frequency of training (low and high, respectively). The sum of the raw AAT points for the five subtests (excluding spontaneous speech) is reported for four different time points. A total of 530 points can be achieved. The classification of aphasia severity is according to the AAT. Entries denoted by ‘x’ indicate missing values. SD, standard deviation.

From the BCI point of view, a combination of frequently missed stimuli, delayed ERP responses and frequent artefacts in patients’ data challenged the decoding system.

### Aachen aphasia test

**Figure 3:**
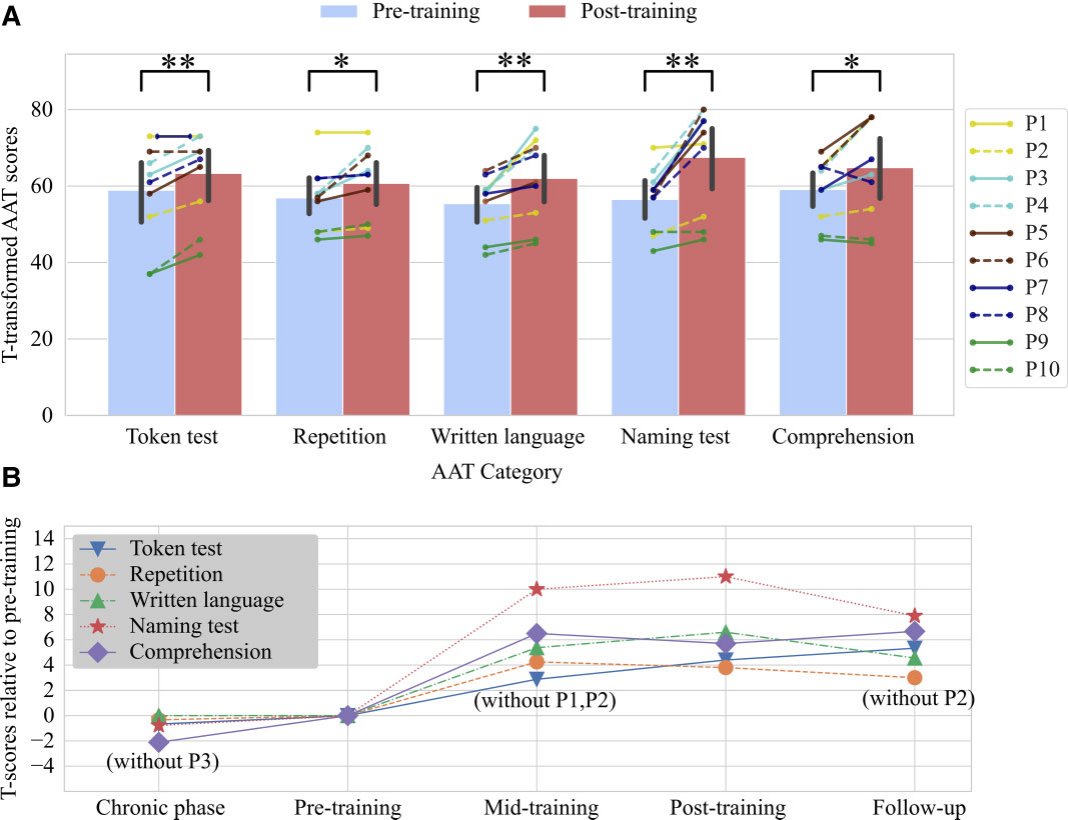
**Clear training-induced improvements of language abilities measured by the AAT**. (**A**) Individual changes and groupwise changes (bars with standard deviations) of different language abilities measured by the T-transformed AAT scores. This transformation normalizes the raw AAT scores such that 10 T-transformed AAT points correspond to 1 SD. Significance was assessed by two-sided paired *t*-tests with the Benjamini–Hochberg correction. The symbol ‘*’ marks *P < *0*.*05 and ‘**’ marks *P < *0*.*01 for the corrected *P*-values. (**B**) The average language performance on the group level at four different time points relative to the pre-training performance. Missing data points are annotated and were excluded from the computation of the averages and the statistical tests.

The main quantifier for training success was the AAT score, as it is standardized and allows for tracking therapeutic success within single patients. The training induced a significant improvement of each AAT subtest in a pre–post-comparison, as shown in Table 2 and Fig. 3. Across all patients and all five subtests, the average effect size is standardized mean difference (SMD) = 0.63 (standard deviation across subjects: 0.36), ranging from 0.38 (repetition subtest) to 1.1 (naming subtest). Patients with mild to moderate aphasia (P2–P8) improved stronger in naming, repetition and writing than patients with moderate/severe aphasia (P1, P9, P10) who mostly showed improvements in the Token test. According to AAT criteria, half of the patients were not aphasic anymore post-training, see Table 3.

A follow-up assessment 3 months after training end showed that these improvements remained stable despite small fluctuations (Fig. 3B). Patient P2 was not available for a follow-up due to an accident unrelated to the training. Comparing follow-up with pre-training performances using a Benjamini–Hochberg-corrected two-sided paired *t*-test, the improvements were still highly significant for the Token test [*t*(8) = 4.208, *P* = 0.001], written language [*t*(8) = 3.957, *P* = 0.001], naming [*t*(8) = 4.318, *P* = 0.006] and significant for comprehension [*t*(8) = 2.731, *P* = 0.03]. The changes in repetition were not significant anymore [*t*(8) = 2.736, *P* = 0.052].

For all patients except P3, we could conduct AAT measurements earlier during the chronic phase, on average 168 ± 149 days prior to training start (Table 3). Improvements during that period were not significant (*α *= 0*.*05), despite all patients except P8 following cSLT at least twice per week, amounting to 30 ± 31 h on average, assuming 40 training weeks per year.

Totalling the number of raw AAT points allows us to directly compare the patients and training-induced effects based on a single value (Table 3). Comparing pre- versus post-training raw points, an average of 49% (range: 11–88%, std = 26.85%) of the maximal possible change^74^ was realized per patient.

### Naming ability, functional communication

The S&V (see Supplementary Fig. 2) revealed a significant pre–post-improvement in the number of correctly named words from an average of 49.0 to 55.7% (Wilcoxon signed-rank test, *Z* = 1, 10 patients, *P* = 0.0039). Changes relative to the pre-training levels are significant also for mid-training (*Z* = 0, nine patients, *P* = 0.0039) and follow-up (*Z* = 1, nine patients, *P* = 0.0078). We observed a significant increase also for semantic and phonological naming scores (see Table 2), but neither for phonological nor semantic access time.

Patients were asked to report on the quality and quantity in everyday language use by the CAL questionnaire.^62^ A two-tailed paired *t*-test revealed highly significant self-reported changes for the quality and quantity of language use, see Table 2. We observed a consistent improvement across all patients, see Supplementary Fig. 3 for individual results.

### Cognitive ability

Before the training, we found that patients showed high pathological performances in cognitive tests which involve language material and partly pathological performances in others (see Supplementary Table 1). Comparing pre- and post-training performances was restricted to five Wilcoxon signed-rank tests (Benjamini–Hochberg-corrected *P*-values at an *α*-level of 0.05) to limit the number of multiple comparisons. The tests showed no significant changes. The results with the uncorrected *P*-values were: digit span (sum forward and backward): *Z* = 19.0, *P* = 0.887 (one tie, one patient could not finish the test); alertness without signal (median time): *Z* = 7.0, *P* = 0.037; alertness with signal (median time): *Z* = 21.0, *P* = 0.507; Go/NoGo (median time): *Z* = 19.0, *P* = 0.734 (one patient could not finish the test); Go/NoGo (total number of errors): *Z* = 20.5, *P* = 0.726 (two ties). For further details, see Supplementary Fig. 4.

### Neuroimaging results

**Figure 4:**
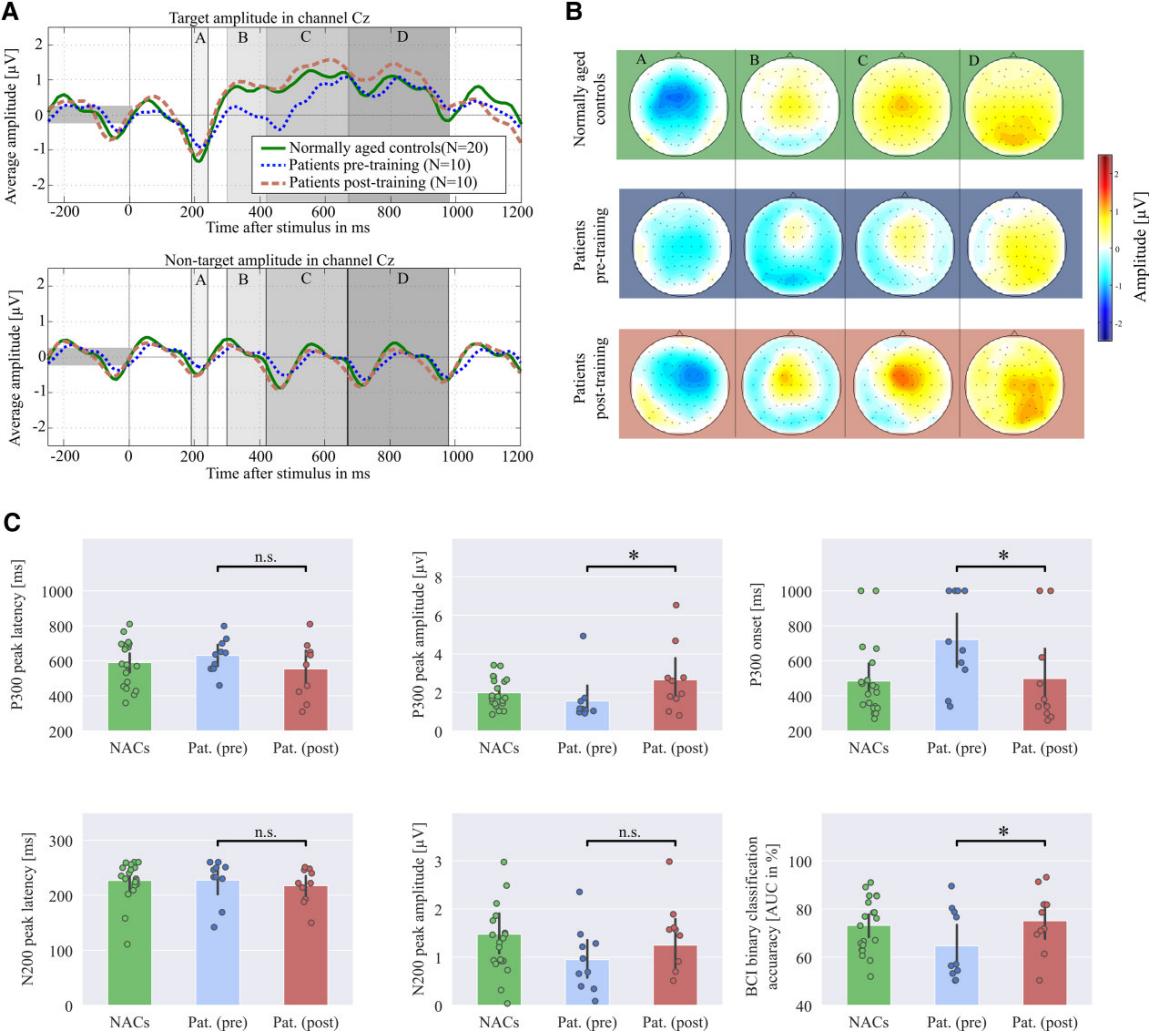
**Stronger and earlier word-evoked P300 responses after the training.** The plots visualize data obtained by ERP offline analysis for patients (pre- and post-training) and for 20 NACs to indicate how healthy subjects process the word stimuli. The ERP responses were evoked by words played with an SOA of 250 ms from six loudspeakers. (**A**) The average target and non-target ERP responses for channels Cz and Fz. (**B**) The spatial distributions of mean target responses within four selected time intervals (in ms relative to stimulus onset): A: (191,240); B: (301,420); C: (421,670); D: (671,800). It can be observed that patients showed P300 and N200 amplitudes lateralized over the right hemisphere before and after the training. However, at post-training, the average ERP time courses and intensities of spatial patterns obtained from patients approximate those of NACs. (**C**) The average (bars with standard deviation) and individual values (dots) for six different metrics. As a result of the training, the P300 amplitudes have increased, P300 onsets have appeared earlier and target versus non-target classification accuracies have increased. Note that no statistical comparisons have been conducted between data of the NACs and patients. All conducted tests are indicated by black bars. n.s., not significant; AUC, area under the receiver-operating characteristic curve; ‘*’ corresponds to *P < *0*.*05.

Three out of six characteristics of the word-induced ERP responses changed significantly (after the Benjamini–Hochberg correction) from pre- to post-training, see Fig. 4C. Patients showed increased P300 peak amplitudes in channel Cz [two-tailed paired *t*-test, *t*(9) = 3*.*35, *P *= 0*.*023], earlier P300 onsets in Cz (Wilcoxon signed-rank test, *Z *= 36, *P *= 0*.*023, two ties) and increased target/non-target classification accuracy expressed by AUC (Wilcoxon signed-rank test, *Z *= 1, *P *= 0*.*023). The N200 peak amplitudes in channel Fz did not change significantly [two-tailed paired *t*-test, *t*(9) = 1*.*25, *P *= 0*.*24] and peak latencies neither changed for the P300 in Cz (Wilcoxon signed-rank test, *Z *= 43.5, *no tie*, *P *= 0*.*15), nor for the N200 in Fz (Wilcoxon signed-rank test, *Z *= 35*.*0, *P *= 0*.*17, one tie).

Concerning patients’ non-verbal auditory oddball ERPs, none of the aforementioned six quantities were significant at the *α*-level of 0*.*05 anymore after correcting for multiple testing, see Supplementary Fig. 5.

**Figure 5:**
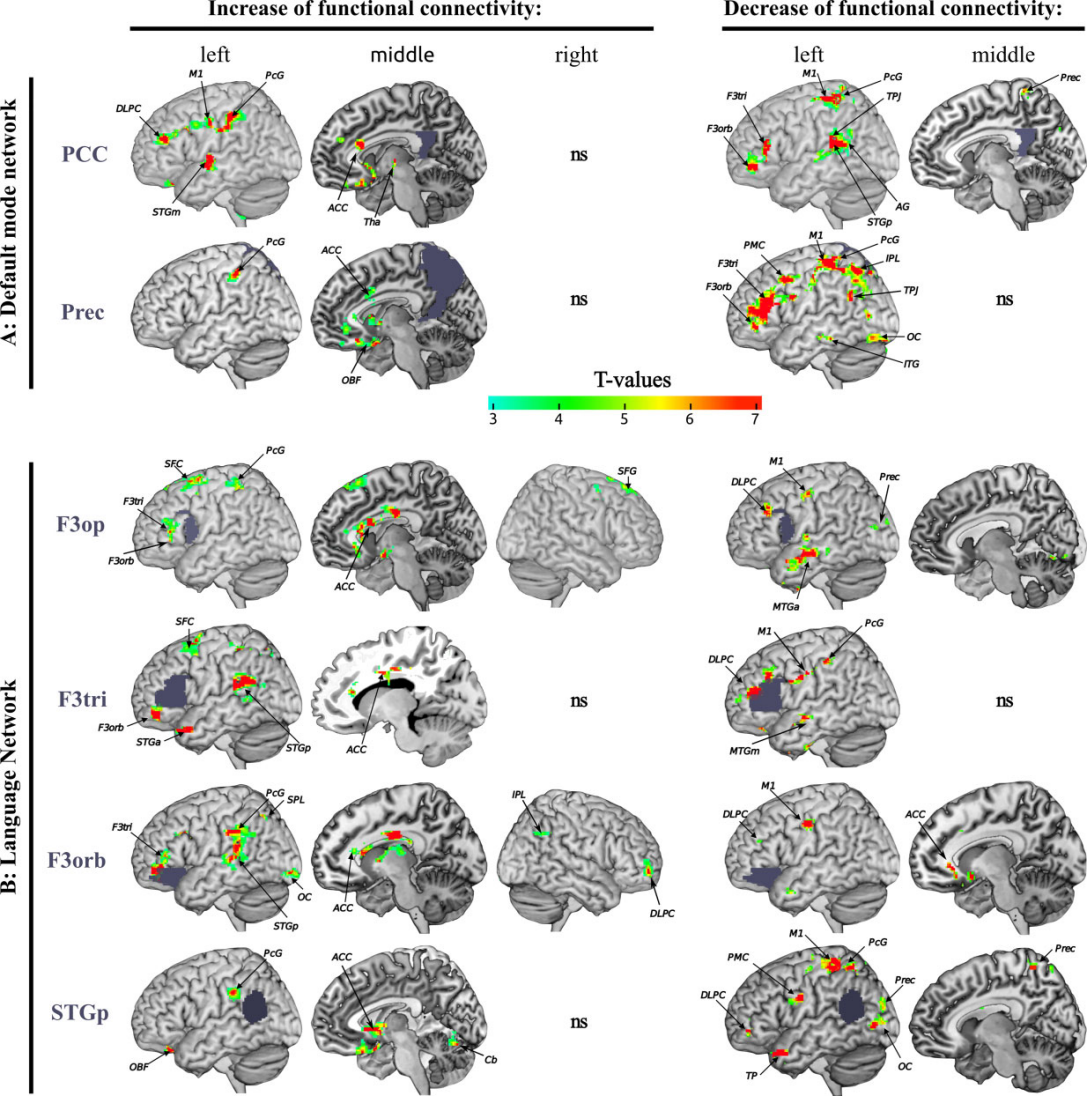
**Training-induced rebalancing of language- and default mode networks.** Pre-/versus post-training changes of rs-fMRI FC are visualized. For every row, the region of interest is indicated by blue-grey colour. The colour bar shows the *T*-values of a paired *t*-test where *T*-values above 3.1 denote a significant change at a family-wise level of *P* < 0.05 after cluster-extent-based thresholding. The FC of the posterior cingulate cortex (PCC) and the precuneus (Prec) with the left postcentral gyrus increase after training. The PCC also shows an increased FC with the left primary motor cortex (M1), dorsolateral prefrontal cortex (DLPC), middle superior temporal gyri (STGm), frontal orbital and anterior cingulate cortex (ACC). The two hubs of the DMN (PCC and Prec) show a decreased FC with the main hubs of the language network—pars triangularis (F3tri), pars opercularis (F3op) and pars orbitalis (F3orb) of the inferior frontal gyrus (Broca’s region) and the posterior superior temporal gyrus (STGp, Wernicke’s region)—as well as with other hubs of the DMN—anterior precuneus, temporo-parietal junction (TPJ), angular gyrus (AG), infero-temporal cortex (ITG), parietal lobe (PL) and occipital cortex (OC). F3op, F3tri and F3orb show increased FC with each other, and also with ACC and STGp. F3orb/tri/op showed decreased FC with MTG, left DLPC, Prec and M1. Wernicke’s region exhibited increased FC with left PL, ACC and cerebellum, and decreased FC with the OC, Prec, TP, premotor and motor cortex (PMC, M1) and DLPC. Non-significant changes are denoted by ‘ns’. Images were generated using mricron (https://www.nitrc.org/projects/mricron).

Comparing the rs-fMRI data before and after the training, we found significant changes of FC of six regions of interest, namely, bilateral posterior cingulate cortex and precuneus—both main hubs of the DMN—as well as pars triangularis, pars orbitalis and pars opercularis of Broca’s area, and left posterior superior temporal gyrus of Wernicke’s area—all main hubs of the language network. The results are summarized in Fig. 5. Changes occurred mainly within the left hemisphere, as reflected by the laterality index ranging from 0.59 to 0.85, see Supplementary Table 2. Decreased FC was observed within the DMN and between the DMN and the language network. Additionally, hubs of the language network showed decreased FC with the dorsolateral prefrontal cortex, the primary motor and sensory cortex. We found increased FC of all regions of interest with the anterior cingulate cortex. Broca’s area showed increased short-range FC within its subcomponents and increased long-range FC with Wernicke’s region. An increase of positive connectivity was observed between the two main DMN hubs and the dorsolateral prefrontal cortex, the primary motor and sensory regions, the parietal lobe, the superior temporal gyrus and the cingulate cortex.

## Discussion

Our study pioneers the use of brain-state-dependent closed-loop feedback to reinforce language-related brain activity in patients with aphasia after stroke. Using a BCI, we strengthened ERP components elicited by a simple language task. The novel training-induced recovery of aphasia, which was sustained, generalized to multiple language aspects and language-specific. A future randomized controlled study will allow us to decide if the BCI-based feedback in fact served as the key factor of training success.

### Feasibility

This study is the first realizing auditory ERP decoding clearly above chance level for aphasic stroke patients and with rapid, non-trivial auditory stimuli.^75^ Remotely related, Kleih *et al*.^76^ applied a *visual* BCI spelling paradigm in multiple sessions with patients with aphasia, but did not assess language improvements.

A rigorous combination of algorithmic improvements, amongst them automatic artefact removal,^77^ session-to-session transfer learning to reduce the need for calibration,^78^ automatic classifier regularization^69^ to cope with a low signal, high dimensionality and noise, rendered our BCI approach feasible (supporting hypothesis H1). We highlight our choice to continuously adapt the classifier.^70^ Besides compensating for non-stationary features, adaptation may have been pivotal for embracing task-solving strategies developed over time.

The training could be executed by all patients and was accepted well, such that all could train frequently and (except P7) also intensively.

### Language improvements

Supporting hypothesis H2, all language metrics (AAT, S&V, CAL) showed medium-to-large improvements when comparing pre- to post-training assessments (Table 2). In comparison with the Cochrane reviews,^5,12^ our training-induced improvements are long-lasting (see Fig. 3). Our average effect size on language is high (SMD = 0.63) considering the values reported for cSLT by Brady *et al.*,^5^ which range from 0.06 to 0.49 for different language competences, and although the 27 studies covered included subacute patients, whose improvement may have been dominated by spontaneous recovery. Note that the cSLT—applied with a similar amount of effective training hours (Table 3)—did not evoke significant changes. Considering that cSLT on average lasted 1.95 h per week, whilst the BCI-based training was delivered 6.26 h per week on average, the intensity of our training might be a factor contributing to the efficiency of the proposed training. However, even comparing our results specifically to a study which focused also on patients with varying severity of chronic aphasia and also administered high-intensity training confirms that our training effect is relatively large. Breitenstein *et al.*^7^ showed an effect size of SMD = 0.23 for verbal communication using high-intensive cSLT (30 h delivered within 3 weeks) compared with low-intensive cSLT (<5 h/week). The authors had reported an effect size of 0.57 in their publication, computed based on the standard deviation of the *differences* instead of the *population*. The former leads to higher effect size estimates in within-subject analyses.^79^ For comparisons, we re-computed the effect size of 0.23 based on the population standard deviation—as recommended by the Cochrane Study Group^5^—using data published by Breitenstein and colleagues. Other studies using the AAT as their primary end-point^19,57,67,80^ reported average AAT improvements of 2–5 T-transformed AAT points (SMD = 0.2–0.5) which stay below those observed for our training.

Importantly, our improvements generalized to all linguistic competencies tested. Notably, we found the strongest effect within the AAT for the naming ability (SMD = 1.1)—confirmed also by the S&V (see also Supplementary Fig. 2)—the competency for which the Cochrane observed the smallest effect (SMD = 0.06).^5^ Moreover, patients also increased significantly in functional communication, described by higher self-reported quality and quantity of language use and spontaneous speech (Table 2). Such a generalization is considered the ultimate goal of aphasia treatment^81^ as it allows producing the greatest effect with the least possible expenditure of training time and effort.^82^ The strong and comprising generalization differentiates our approach from most other trainings.^5,6^ We attribute it to our design decision not to implement a deficit-oriented training, e.g. not to reinforce based on N400 or P600 features as markers of semantic^26,83^ or syntactic processing,^28^ respectively. Instead, we utilize an elementary language task, which even our patients with severe aphasia could perform and thus could profit from the BCI’s feedback. We found that training up a basic language competence consequently facilitated higher language skills including production and written language skills. This finding may be explained by basic and higher language skills partially sharing the same networks, by interwoven networks for language production and comprehension^33–38^ and by areas shared between writing and oral spelling of object names.^84^

Moreover, note that 50% of patients who displayed stable mild aphasia despite a regular cSLT were classified as non-aphasic by the post-training AAT (Table 3). The AAT profile determines the clinical picture and severity of aphasic syndromes beyond a mere statistical test. The classification ‘no aphasia’ means that the resulting clinical picture did not lead to a specific syndrome and the subtests’ performance could not be distinguished from those of healthy subjects. Whilst patients might remain with some communication deficits that typically become evident in the form of phonological or semantic paraphasias or word-finding disorders in spontaneous speech (reflected by the discrepancy between AAT results in Fig. 4 and CAL results in Supplementary Fig. 3), a change from ‘aphasia’ to ‘no aphasia’ is a clear, substantial success. Considering that when AAT subsets are pathological, their constellation fits the clinical picture of language disorders characterized by non-fluent production (Broca's aphasia) or limited vocabulary access (anomia), significant pre–post-changes even in a single subset are remarkable from a clinical point of view. Although not standardized, the additional CAL, AAT spontaneous speech and S&V tests address relevant language skills, and observed deficits are recommended to be treated.^8,9^ Thus, the sustained language improvements documented by our study can be considered very promising and we hope we can replicate them in the future by a larger randomized controlled study.

Supporting H3, our training is associated with a recovery from aphasia, but not from non-linguistic deficits (Table 2). Thus, language recovery probably cannot be explained by improvements of non-linguistic competencies alone.

### Possible mode of action

The novel BCI training has enhanced ERP classification accuracies (Fig. 4), a surrogate for how well a patient’s brain could discriminate between target and non-target words. Concerning ERP components, only the P300 showed significant modifications after the training (Fig. 4). This component is known to predict recovery from aphasia^29^ and increased P300 peak amplitude and earlier onset generally indicate that the task may have become easier.^85^

According to event-categorization and template-matching models,^85^ as well as to the context-updating hypothesis,^86^ the P300 reflects the integration of bottom-up and top-down processes. More specifically, P300 reflects integration processes and its latency corresponds to the stimulus evaluation time.^87^ The post-training P300 characteristics (Fig. 4A) indicate that the BCI feedback may have positively influenced integration processes, i.e. via faster stimulus evaluation or an individually strengthened sensory–motor processing of language. Interestingly, patients reported the use of inner speech as a successful strategy. Considering that the internal and external speech within the Levelt model highly interacts at the neuronal level,^33^ we postulate that the integration of bottom-up and top-down and internal speech processing may contribute to explaining the mode of action of the BCI training.

We assumed that the P300 would also reflect attention and working memory within the event-categorization network.^86^ For the non-linguistic auditory oddball task, however, our training neither induced substantial P300 modifications nor improved in classification accuracy (Supplementary Fig. 5), probably as our training has reinforced word-evoked ERPs only. Together with the absence of significant cognitive improvements, this finding suggests that (i) our approach does neither substantially train working memory nor attention and (ii) the observed language benefits cannot directly be explained by improvements of these two competencies. Overall, the context-updating hypothesis—involving closing the loop between bottom-up processing, top-down control and propositional language—delivers a better explanation of our results.

Our FC results support this hypothesis to some extent. The post-training data showed an increased short-range FC within the different parts of Broca’s area, consistent with Broca’s dependency upon local processing.^88^ Strengthened long-range FC between left inferior frontal gyrus and temporo-parietal regions (Fig. 5) might reflect the reinforcement of stroke-spared connections of the language network.^50^ These regions are interconnected by the dorsal and ventral pathways.^42–46,89^ Particularly, a strengthening of the dorsal system may indicate improved sensory–motor integration. The dual-loop system is considered the anatomical framework for basic building blocks like word perception and production^46,90^ as well as for higher skills such as syntax processing.^44^ Conversely, a strengthening of the dual loop may explain the observed improvements, which were not limited to basic word processing, but became apparent also for higher linguistic skills. The reported high interaction between phonological and orthographic processing, both at a cognitive and anatomical level^35,89,91^ may explain why training with phonological complex stimuli has improved orthographic skills in our patients.

We also observed significant FC changes within the DMN and an altered interaction between DMN and the language network. The DMN typically responds in a task-dependent manner to increased effort.^92^ We found (Fig. 5) that main regions of the DMN like the posterior cingulate cortex and the left precuneus^93^ significantly decreased their FC with (i) other DMN regions; (ii) with brain regions functionally related to the language system; (iii) the decision-making system localized in the left dorsolateral prefrontal cortex^94^; (iv) regions responsible for the execution and attentional monitoring of spatial behaviour (generally related to parietal lobe, primary motor and sensory regions) and (v) the saliency network (cingulate cortex).^95^ Given our current limited data and the chosen experimental design, we refrain from stating a causal relationship between FC changes and language recovery. However, the FC findings are in line with the proposal that cognitive functions are linked to dynamic, anticorrelated networks^95,96^ and that language not only depends on the interactions within the dual-loop but also on those between this language network and domain-general networks.^97^ In stroke patients with aphasia, Geranmayeh *et al.*^50^ observed an upregulation of DMN activity, i.e. the intact domain-general systems for cognitive control and attention. They associated the combination of higher activity of the language network and lower activity in the DMN with improved speech production. Our resting-state results support the hypothesis that the combination of a strengthened language network and a downregulated DMN, i.e. a (re-)balancing of the interaction between the lesioned network and the DMN, may explain recovery from aphasia.

### The role of the BCI

Importantly, BCI feedback can be pinpointed to reinforce language processing instead of motor execution, e.g. word production or gesticulation, which could be reinforced also by cSLT.

If an intact language network integrates sub-processes by synchronization mechanisms, speeding up sub-processes involved in target versus non-target recognition should be beneficial. Working with an EEG-based BCI allowed us to address timing aspects: target and non-target words were presented in a quick sequence and increasing the presentation speed as long as target/non-target classification accuracy indicated good performance kept the task challenging throughout the training. As patients have been described to show delayed processing compared with controls—indicated by prolonged P300 latency upon auditory oddball tones^29,98^ and prolonged reaction times upon phoneme stimuli in a mismatch negativity paradigm^99^—and as we have observed training-induced P300 onset reduction, we argue that training up the language processing speed supports recovery.

It would be interesting to investigate, if our training makes the word-evoked ERP responses more similar to those of healthy controls, as suggested in Fig. 4. Unfortunately, our protocol has not been designed for this purpose as the NACs and the patients were not matched for sex or other potential confounding factors. A specifically designed, novel study is required to answer, if sex can influence our word ERP characteristics and the training’s efficiency, or if our training can recover the shorter ERP latencies and larger amplitudes of healthy controls, independently of sex.

To cope with the task, patients were suggested to exploit potentially beneficial strategies, e.g. the use of inner speech, to imagine the target or to focus more on either spatial (loudspeaker condition only) or phonological cues. We chose not to require patients to follow a specific strategy, as no single one could suit well the high variability of patients’ capabilities. Observing more discriminative ERP responses over training sessions, we assumed that a patient had discovered an individually successful strategy, which enabled him/her to solve the word recognition task. Whilst a strategy could not necessarily be described and some patients were not even aware of it, our goal was to positively reinforce any such successful strategy by BCI feedback. Technically, we supported this by a machine learning pipeline capable to train an individual classifier on any target versus non-target ERP feature differences observed, as long as basic plausibility requirements regarding the neural origin of ERPs were met. Note that in particular, the use of a BCI allowed reinforcing strategies early on during the training and independently of behaviourally recognizable improvements.

Although not observed in our study, BCI feedback could potentially train up an undesirable signal source. This drawback is discussed together with other potential limitations in Supplementary Section 9.

Moreover, also the rigorous implementation of the latest design recommendations for closed-loop training in BCI^100^ may have contributed to the observed high training efficiency. See Supplementary Section 10 for details.

## Summary

The proposed novel BCI-based training approach has resulted in a medium to large and generalized language improvement independent of the initial severity of aphasia. The induced recovery is associated with a strengthening of the language network, the rebalancing of networks and by improved timing and synchronization in combination with high training efficiency. Considering the current debate on whether neuroplasticity explains therapy-induced improvement, our study contributes evidence that the reinforcement of selected brain activity can be beneficial for cognitive rehabilitation.

## Supplementary Material

fcac008_Supplementary_Data

## Abbreviations

AAT =: Aachen Aphasia Test
AUC =: area under the receiver-operating characteristic curve
BCI =: brain–computer interface
CAL =: communicative activity log
cSLT =: conventional speech and language therapy
DMN =: default mode network
ERP =: event-related potential
FC =: functional connectivity
LDA =: linear discriminant analysis
NACs =: normally aged controls
ROI =: region of interest
rs-fMRI =: resting-state functional MRI
SD =: standard deviation
SMD =: standardized mean difference
SOA =: stimulus onset asynchrony
S&V =: Snodgrass & Vanderwart naming test
TAP =: test of attentional performance
