# Supplementary material for: Aphasia recovery by language training using a brain–computer interface: a proof-of-concept study
Source: Brain Commun. 2022 Feb 8;4(1):fcac008. doi: 10.1093/braincomms/fcac008 (PMC8846581; doi:10.1093/braincomms/fcac008)
Supplement: fcac008_Supplementary_Data [file fcac008_supplementary_data.pdf]

Supplementary Information

Contents

1 Snodgrass & Vanderwart naming test: implementation and evaluation 3

2 Cognitive assessments and results 5

3 EEG data: recording, processing and dynamic stopping 8

4 Stimulus material 8

5 Single session with normally aged controls 9

6 Adaptation of task difficulty 9

7 fMRI laterality indices 9

8 Standardized mean difference via Hedges' g 9

9 Potential risks 10

10 Training efficiency by design 10

11 Inclusion and exclusion criteria 11

12 Different levels of BCI-based feedback 11

13 Non-verbal auditory oddball results 12

14 Patient-wise results for the communicative activity log (CAL) 13

15 Laterality indices for different seed regions in the resting-state fMRI analysis 13

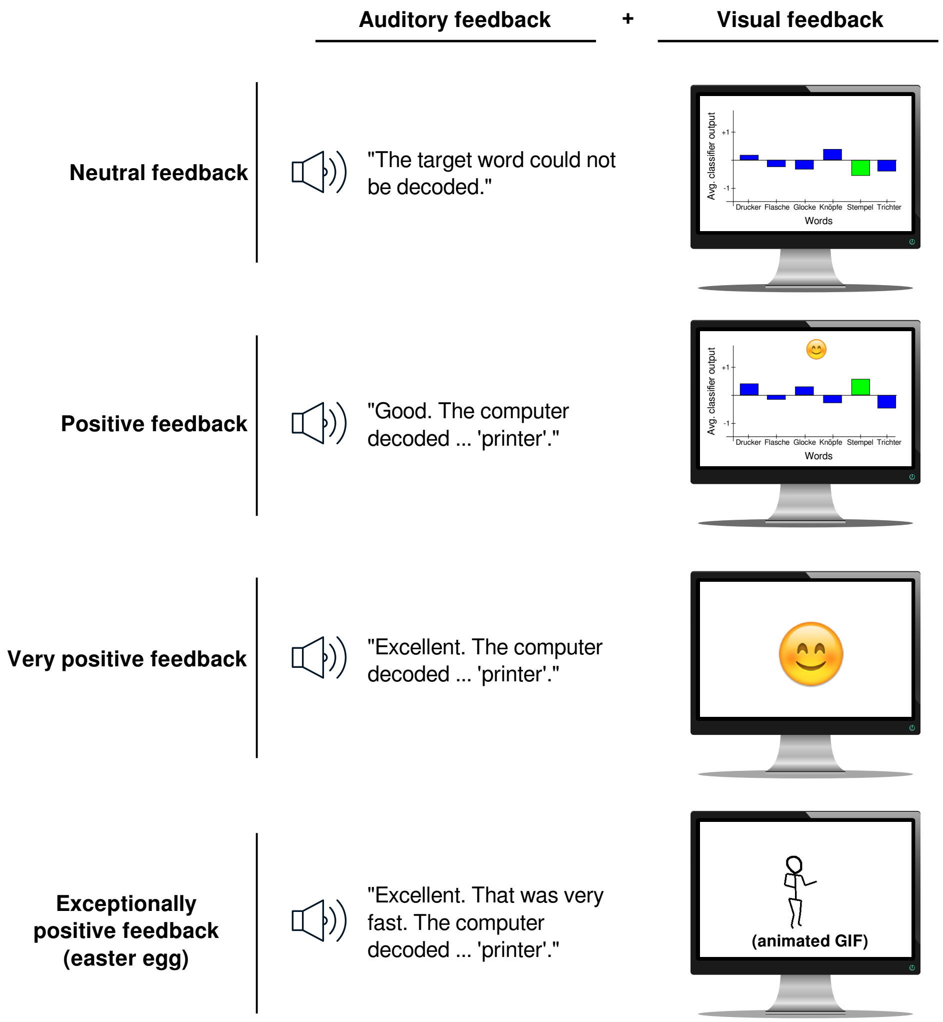

Supplementary Figure 1: **Four different levels of auditory and visual feedback could be provided after a BCI-training trial.**

1 Snodgrass & Vanderwart naming test: implementation and evaluation

The Snodgrass & Vanderwart naming test was implemented using the Presentation software. For every item, the corresponding image was presented to the participant, who had to name the item. A recording of the naming was stored. We programmed a custom-made Python application to evaluate these sound recordings. It supports the human rater by playing one recording at a time and requiring the rater to provide various metrics:

Correctly produced? A word counted as correctly produced also if the word could be produced after initial uncertainty, self-improvement or search behavior, or if a reasonable synonym was produced.

Semantic score. The semantic score addresses the patient's ability to access the right meaning of the word. It was rated on a scale from 0–4 points with the following levels:

- **4P:** Correct word or appropriate synonym/dialect.
- **3P:** Similar to **4P** but with search behavior, self-improvement or uncertainty.
- **2P:** An appropriate description, umbrella term, neologism, description in foreign language, etc.
- **1P:** An inappropriate but related description, umbrella term, etc.
- **0P:** No correct meaning could be detected.

Phonological score. The phonological score addresses the patient's ability to produce the right pronunciation of the word. It was also rated on a scale from 0–4 points with the following levels:

- **4P:** Correct production of the word (dialect was accepted, too).
- **3P:** Similar to **4P** but with search behavior, self-improvement or uncertainty.
- **2P:** At least two-thirds of the phonemes are correctly produced in the best version of the word.
- **1P:** At least one-third of the phonemes are correctly produced in the best version.
- **0P:** The target word could not be detected.

Semantic delay. In addition, we assessed the time until the patient first got access to the right meaning of the word. This metric was only evaluated if the semantic score was **2P** or better.

Phonological delay. We also assessed the time until the patient first correctly produced the word. This was only evaluated if the semantic and phonological scores were **3P** or more.

All recordings of a patient were evaluated by the same single rater. All raters were employed by the University Medical Center Freiburg or the University of Freiburg and were part of the project team. For the first eight patients, they evaluated the naming tests one by one with potential knowledge of the session timing (pre/mid/post/follow-up). For patients P9 and P10, we shuffled the words from the different sessions during the evaluation stage such that the rater could not know the assignment between recordings and sessions.

The rate of correctly produced words of the Snodgrass & Vanderwart test was assessed at four time points, see Figure 2. For details of the other Snodgrass & Vanderwart metrics, please refer to the main text.

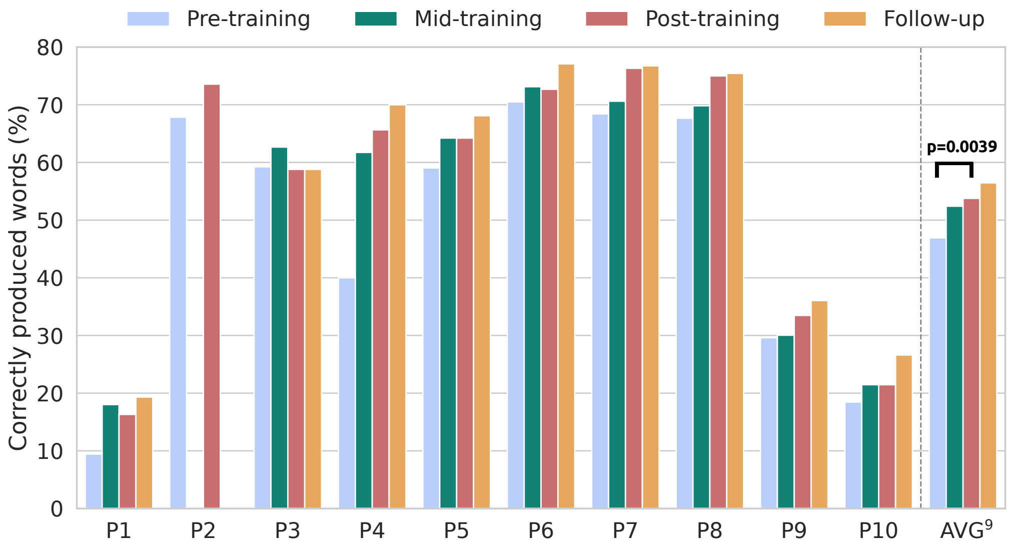

Supplementary Figure 2: **Training-induced improvement of naming ability as measure by the Snodgrass & Vanderwart test.** The average values (AVG[9]) are computed without patient P2 to account for the missing data of the mid- and follow-up assessments. The average pre-post training improvement of the number of correctly produced words is significant ($\alpha = 0.05$, paired t-test).

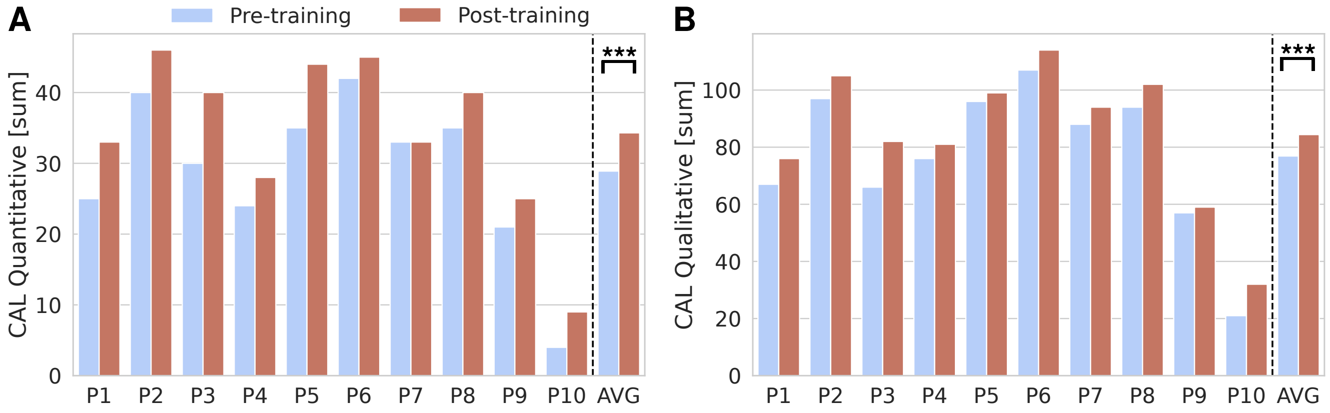

Supplementary Figure 3: **Self-reported everyday communication measured by the communicative activity log (CAL)**. Subplot **A** shows the quantity and subplot **B** shows the quality of language use in everyday situations as reported by the patients. Significance was assessed with a two-sided paired t-test where *** indicates $p < 0.001$.

2 Cognitive assessments and results

Patients underwent a series of cognitive tests before and after the training. In this section, we (1) provide additional information about these tests, (2) show that patients have a pathological performance before the training and (3) provide evidence that patients did not improve in these tests over the training. We start with a short description of the different cognitive tests.

- **Digit span.** This test examines the short-term verbal working memory ability (Baddeley, 1992). Participants are asked to repeat numbers in correct forward or backward order immediately after their oral presentation.
- **Semantic and phonological fluency.** In these four different tests, participants have to either name as many animals or jobs as possible in a limited time (semantic fluency test) or as many words starting with S or with K as possible (phonological fluency test). The word fluency test is called *Regensburger Wortflüssigkeits-Test* (Aschenbrenner *et al.*, 2000).
- **Corsi span.** In this test, patients have to mimic a researcher as he/she taps a sequence of up to nine identical spatially separated blocks (Kessels *et al.*, 2000). It assesses visuospatial short term working memory.
- **Reaction time to a visual stimulus (alertness).** In this test, patients have to press a button upon perceiving a visual stimulus. The median of the reaction time is the quantity of interest. The test is subdivided into two subtests: The first one assesses tonic alertness, which is defined as the ability to maintain a high level of responsiveness in anticipation of a visual test stimulus. The second one assesses phasic alertness, where the participant is warned by a beep tone presented prior to the visual target stimulus. This test is part of the test of attentional performance (TAP) (Zimmermann and Fimm, 2002).
- **Selective and divided attention.** Two forms of attention are investigated by this test. The first one addresses selective attention assessed by a visual Go/NoGo task (pressing a button only if a cross appears on the screen, but not if a plus symbol appears) in terms of reaction time and the number of errors. The second tests for divided attention and includes the simultaneous reactions to a visual and an auditory task. Both tests are part of the TAP (Zimmermann and Fimm, 2002).
- **Flexibility.** This tests the competence to gear attention again and again to different relevant aspects of a situation. Patients see a letter and a number side by side. For each trial, patients receive an alternating task: first, they either need to indicate the side of the letter's or the number's appearance by pressing a left or right key. In the following trial they need to indicate the position of the other symbol. This test is also part of the TAP (Zimmermann and Fimm, 2002).

Given the characteristics of the modified auditory AMUSE paradigm, we assume that our online training would rather not influence visual memory, spatial working memory and flexibility, although we know that these aspects are known to be important for the recovery from aphasia. Thus the statistical analyses for training-induced effects upon cognitive skills were limited to five quantities only, which reflect changes of attention, verbal working memory and alertness.

The results of the cognitive tests as measured *before* the training are provided in Table 1.

Supplementary Table 1: Pre-training percentile ranks for the 10 chronic patients with aphasia in different cognitive tests.

	**Digit span**		**Semantic fluency**		**Phonological fluency**		**Corsi span**		**Alertness**		**Attention**		**Flexibility**
patient	forward	backward	animal	jobs	S-words	K-words	forward	backward	tonic	phasic	divided	selective	median
1	**x**	**x**	**<2**	**2**	**5**	17	**<2**	**<10**	58	27	**<1**	42	**10**
2	20	**12**	**4**	**4**	**<1**	**<1**	**7**	70	27	**8**	**8**	**10**	**3**
3	**<2**	**2**	**2**	**6**	**<1**	**<1**	**<7**	**10**	46	46	**x**	**x**	**x**
4	**5**	27	**11**	**5**	**5**	**5**	**<2**	**<10**	**5**	**4**	**<1**	**<1**	**x**
5	**2**	**2**	**4**	**3**	**<1**	**<1**	27	22	50	42	>31	90	46
6	**2**	**5**	**5**	**2**	**2**	**2**	**13**	**13**	24	**14**	>31	79	**14**
7	**2**	**2**	**2**	**<3**	**<1**	**<1**	50	31	**14**	**8**	**8**	16	31
8	67	**12**	28	63	**1**	**1**	95	85	**2**	**3**	>31	**14**	21
9	**<2**	**<5**	**<1**	**<1**	**<1**	**<1**	32	40	79	79	27	**12**	**8**
10	**<2**	**<5**	**<1**	**<1**	**<2**	**<2**	**<5**	**11**	42	31	**x**	**10**	**x**

Legend:
x: Patient was not able to perform the test.
<: Performance was worse than this percentile rank.
Pathological performance (defined as a performance below the 15th percentile) or the inability to conduct the test at all, are marked with bold entries and are shaded in gray.

To assess if training-induced changes also affect cognitive functions such as verbal memory, alertness or selective attention, we statistically evaluated a subset of the cognitive tests. Its number was restricted to five to limit the number of multiple comparisons. For all significance tests, we took a two-sided Wilcoxon-signed rank sum test to account for the non-Gaussian distributions. The results with the uncorrected p-values are shown in Figure 4 and the following list:

- Digit span (sum forward and backward): Z=19.0, p=0.887, one tie, one patient could not finish the test.
- Alertness without signal (median time): Z=7.0, p=0.037.
- Alertness with signal (median time): Z=21.0, p=0.507.
- Go/NoGo (median time): Z=19.0, p=0.734, one patient could not finish the test.
- Go/NoGo (total number of errors): Z=20.5, p=0.726, two ties.

The only training-induced change significant at an uncorrected α-level of 0.05 was the decrease in the median reaction time in the alertness task. After correcting for multiple testing, none of the changes remained significant, see Figure 4.

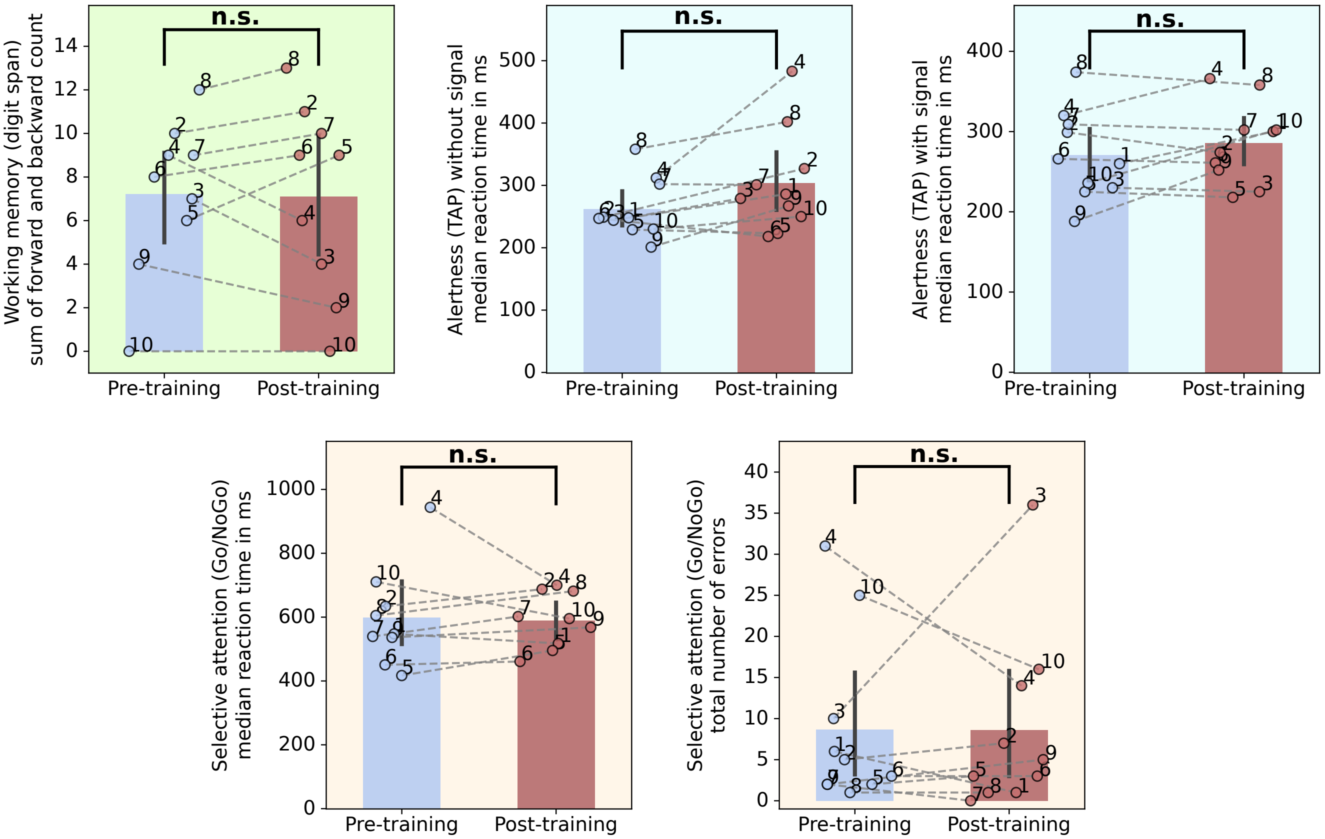

Supplementary Figure 4: **Cognitive test results for tasks evaluating working memory, alertness and attention.** Single points indicate individual patients. The p-values have been corrected for multiple testing with Bonferroni-Holm correction. **n.s.** = not significant, **TAP** = test of attentional performance.

3 EEG data: recording, processing and dynamic stopping

Brain activity was recorded and amplified by a multichannel EEG amplifier (BrainAmp DC, Brain Products) with 63 passive Ag/AgCl electrodes (EasyCap) during the offline EEG-sessions before and after the training and with 31 passive electrodes during the online training sessions. The channels were placed according to the 10-20-system referenced against the nose and grounded at channel AFz. The sampling rate was 1 kHz. Impedances were always kept below 20 kΩ. Eye signals were recorded by electrooculography with an electrode below the right eye.

The data was bandpass filtered to [0.5, 8] Hz for classification and to [0.5, 12] Hz for visualization of averaged ERP epochs using a third-order Chebyshev Type II filter before downsampling it to 100 Hz. To extract ERPs, the EEG signals were epoched between -200 ms and 1200 ms relative to the stimulus onset. A baseline correction was performed relative to the data segment within the interval [-200, 50] ms. As features for classification, the average amplitudes were extracted in the ten intervals [80, 150; 151, 210; 211, 280; 271, 350; 351, 440; 450, 560; 561, 700; 701, 850; 851, 1000; 1001, 1200] ms relative to the stimulus onset. For an online session, this resulted in 310-dimensional feature vectors (31 channels) per stimulus epoch. From offline sessions, we obtained 630-dimensional feature vectors using the same preprocessing and feature extraction procedure but 63 instead of 31 channels.

During the online sessions a so-called dynamic stopping strategy (Schreuder *et al.*, 2013) could be triggered after the presentation of at least 42 words, as soon as a one-sided Welch's t-test of the collected classifier outputs indicated a significant difference between the target word and the best non-target word. The significance level was set to 0.05 and could be lowered further by the experimenter as one possibility to adapt the overall task difficulty over time.

4 Stimulus material

The stimulus material consisted of 6+8 German sentences (with the last words missing) and the corresponding bisyllabic words (presented separately). The initial 6 sentences and words were the following:

```
Die neue Tonerkartusche steckt schon im ... Drucker.
In der Getränkekiste steht noch eine volle ... Flasche.
Am Ende der großen Pause läutet die ... Glocke.
Die Jacke des Kapitäns hat goldene ... Knöpfe.
Zur Beglaubigung benutzt die Sachbearbeiterin einen amtlichen ... Stempel.
Zum Nachfüllen des Motoröls nimmt man am besten einen ... Trichter.
```

These 6 sentence/word pairs were used with patients during the pre- and post EEG sessions without feedback, and during the first training sessions with feedback. For the single session with healthy controls, only these six pairs were used. For patients we prepared eight additional sentence/word pairs as candidates to replace initial sentence/word pairs, if the difficulty of the task needed to be increased. In our study, however, these were rarely used:

```
Weil es kälter wird, schließt er den Reißverschluss seiner ... Jacke.
Um Wasser zu holen, schöpfte man es früher aus einem tiefen ... Brunnen.
Das Schutzblech am Fahrrad klappert, es fehlt eine ... Schraube.
Im Alter benötigt man zum Lesen oft eine ... Brille.
Um aufs Dach zu gelangen, benutzt der Schornsteinfeger eine lange ... Leiter.
Das Regenwasser wird aufgefangen in einer großen runden ... Tonne.
Auf dem Herd stehen Pfannen und ... Töpfe.
Um den Hals trägt sie gerne ihre goldene ... Kette.
```

5 Single session with normally aged controls

The normally aged controls (NACs) underwent a single session only and did not receive any feedback. Words were presented only at a single, fixed SOA of 250 ms. In addition to the condition using six loudspeakers and the headphone condition, the NACs also performed a third condition where all stimulus words were presented from a single loudspeaker. Within each NAC's single session, the three conditions were pseudo-randomized between 18 runs, with a longer break every six runs and shorter breaks between runs. While for patients, the word conditions were not changed within any of their online training sessions, NACs had to cope with 17 switches between the three conditions within their single offline session. The data of the NACs' 6 loudspeaker condition is used for comparisons with patients. Data obtained from NAC's during the two other conditions will not be reported on.

6 Adaptation of task difficulty

The following adaptations could be applied: first, single sentence/word combinations could be replaced to account for saturating classification performance for that combination. At most, one sentence-word combination was replaced between sessions, and only if a saturated performance had been observed over at least two sessions. Second, the initial loudspeaker condition could be replaced by the more demanding headphone condition. This adaptation could be performed only after 15 of the foreseen total 30 hours of active training time (typically after approximately 7 sessions). Due to the strongly increased task difficulty, this adaptation was not applied for all patients. Third, the SOA could be altered. Typically, patients started using a slower SOA (e. g. 350 ms) within a condition (e. g. loudspeaker condition) and could switch to a more demanding one (e. g. 250 ms) after a few sessions. When switching to the more demanding headphone condition, however, the SOA initially had to be increased again. Fourth, the significance threshold for the dynamic stopping mechanism (see supplementary section 3) could be adapted to influence the frequency of early stops and the resulting very positive or exceptionally positive feedback see Figure 1.

7 fMRI laterality indices

For fMRI data, laterality indices (LI) were computed on the statistical maps as follows

$$\mathrm{LI} = \frac{N_L - N_R}{N_L + N_R} \tag{1}$$

where N_L and N_R are the number of voxels with significant functional connectivity changes in the left and right hemispheres, respectively, so that LI $= +1$ for exclusively left-hemispheric changes and LI $= -1$ for exclusively right-hemispheric changes.

8 Standardized mean difference via Hedges' g

Hedges' g is one possibility to define the standardized mean difference (SMD). It is defined as the quotient of the differences between the means and the pooled standard deviation (Lakens, 2013)

$$g_s = \frac{\mathrm{Mean}(\mathbf{z}_{\mathrm{post}}) - \mathrm{Mean}(\mathbf{z}_{\mathrm{pre}})}{\sqrt{\frac{\mathrm{SD}(\mathbf{z}_{\mathrm{pre}})^2 + \mathrm{SD}(\mathbf{z}_{\mathrm{post}})^2}{2}}} \cdot \left(1 - \frac{3}{8 \cdot N - 9}\right) \tag{2}$$

where $\mathbf{z}_{\mathrm{pre}}$ and $\mathbf{z}_{\mathrm{post}}$ denote the test results before and after the training for N patients, respectively.

9 Potential risks

Our feedback training approach bears the potential risk to train up the source of a signal which displays discriminative information about the target vs. non-target classification problem, even if this source is not intended to be trained. The main reason for this is our adaptive decoding approach. It unfortunately cuts both ways: it not only allows us to flexibly detect and exploit stroke-induced non-standard brain responses on the one hand (which is more than welcome to compensate for inter-subject variability and training-induced non-stationarity of features), but may also be deceived by eye blinks or motor-related potentials, that we do not intend to reinforce. Such a situation occurred during the training of patient P2 who started to use motor-related potentials in addition to ERPs upon target appearances. Thus, we decided to abort the training after 24 hours. While we are aware that this risk cannot be fully excluded, we limited its impact by visual inspection of the obtained data and the linear classification model after each session, and by close observation of patients' activities during the session.

10 Training efficiency by design

Another reason for the high training efficiency may be the rigorous implementation of the latest design recommendation for closed-loop training that has been found essential for quick user learning in BCI applications (Lotte *et al.*, 2013). This comprised (1) providing graded informative feedback with information about runner-up words, (2) a personalized and variable degree of task difficulty, considering a patients abilities while maintaining high training pressure (individualized SOA and stimulus presentation scheme), and (3) maximizing the number of trials executed per session by making use of a dynamic stopping procedure in each trial (Schreuder *et al.*, 2013). In addition, we kept a high training intensity by maximizing the duration per session (considering the patient's stamina) and by densely scheduling sessions to quickly accumulate 30 hours of effective training.

Supplementary Table 2: **Details on training times.**

	Training duration [hours]	Training duration [days] (= number of sessions)	Training hours / week	Whole training period [days]	Extraordinary breaks, reason
P1	35,30	15	7,06	42	16 days due to illness after session 14
P2	24,06	11	6,56	15	
P3	29,97	14	6,42	22	
P4	29,70	17	5.24	30	
P5	29,27	11	7,98	22	
P6	29,98	13	6,91	25	
P7	30,20	25	3,6	74	31 days of holidays between blocks 1 and 2
P8	29,53	15	5,9	27	
P9	30,37	15	6,07	28	
P10	30,05	13	6,93	24	
			AVG: 6,267		

11 Inclusion and exclusion criteria

For patients, the inclusion criteria were:

- a single left-hemispheric ischemic stroke affecting the left middle cerebral artery (additional infarction of anterior cerebral artery and other arteries are acceptable)
- time point of stroke at least six months prior to start of training
- presence of aphasia (confirmed by Aachener Aphasie Test, AAT)
- age between 18-80 years
- German as native language
- sufficient cognitive abilities to comply with the study requirements

Exclusion criteria were:

- a bilateral stroke
- hemorrhagic stroke
- structural brain or skull lesions (tumor, trauma, high cerebral microangiopathy) reported or visible in the MRI
- high cerebral artery stenosis
- history or current diagnoses of other medical, neurological or psychiatric disorders interfering with participation or data analysis
- severe hearing deficit
- vision loss
- factors hindering fMRI or EEG acquisition, e.g., medication known to affect cognition or vigilance
- early bilingual patients
- professional musicians
- left-handedness

For normally aged controls (NACs) the same exclusion criteria were applied as for patients.

12 Different levels of BCI-based feedback

The four levels of BCI-based feedback, ranging from neutral to exceptionally positive, are illustrated by Figure 1.

All feedback was provided in German. The translations provided in Figure 1 are for the reader's convenience. The patient received different feedback levels based on how clearly the classifier responses for the target word differed from the non-target word responses. Neutral feedback was given if a non-target word (indicated by a blue bar) had a higher average classifier output compared to that of the target word (indicated by a green bar). Positive feedback was given if the classifier output for the target word was larger than for all non-target words, but only by a small margin. Very positive feedback was given if a dynamic stopping event was triggered. This had been the case if a one-sided Welch test between the target word and best non-target word yielded a p-value of below 0.05 (or less, if the α-level was changed to make the training more difficult). Exceptional feedback was given with a 25 % chance, if a dynamic stopping event was triggered already at the earliest possible time point (after 42 word presentations).

13 Non-verbal auditory oddball results

Patients as well as normally-aged controls (NACs) performed two non-verbal oddball runs at the start of each session. Each run took around five minutes and consisted of a pseudo-randomly interleaved sequence of 50 high-pitched target tones and 250 low-pitched non-target tones with tones presented using a stimulus onset asynchrony of one second and from a single loudspeaker positioned at the front-right position. Patients were instructed to attend the target tones while ignoring the non-target tones. In the following, we present a statistical analysis of the oddball ERP responses obtained in a single session of the NACs and compare it against the oddball ERP responses of patients obtained during offline EEG sessions before and after the training. The following list shows the detailed test results, and Figure 5 provides an overview of the results with the uncorrected p-values:

- P300 peak latency (ms): two-sided Wilcoxon-signed rank test: Z=25.0, p=0.846.
- P300 peak amplitude (μV): two-sided paired t-test: t(9)=-0.456, p=0.659.
- P300 onset (ms): two-sided Wilcoxon-signed rank test: Z=9.0, p=0.208, #ties=2.
- N200 peak latency (ms): two-sided Wilcoxon-signed rank test: Z=30.5, p=0.343, #ties=1.
- N200 peak amplitude (μV): two-sided paired t-test: t(9)=-0.652, p=0.531.
- BCI classification accuracy (AUC): two-sided Wilcoxon-signed rank test: Z=49.0, p=0.027.

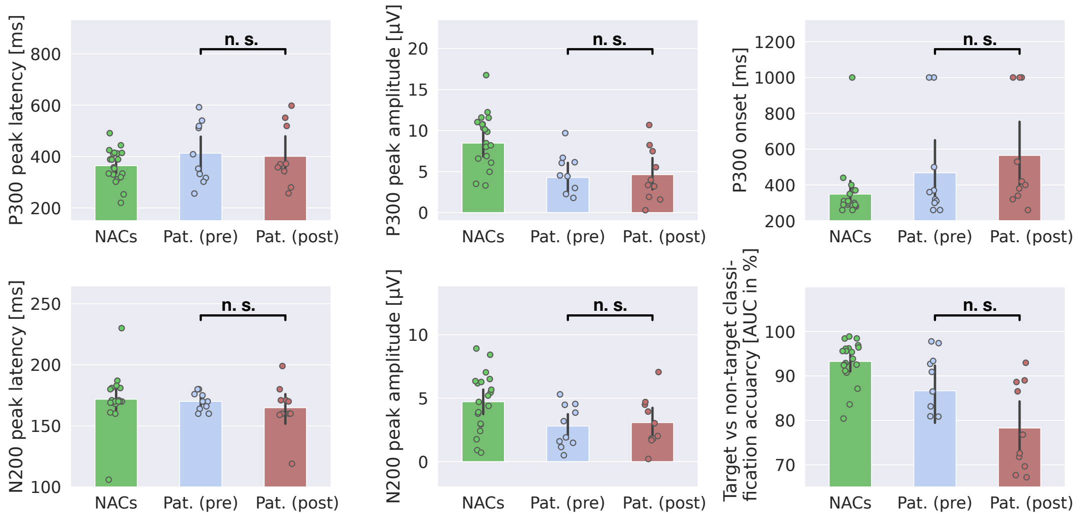

Supplementary Figure 5: **No significant training-induced differences of ERPs evoked by a non-verbal oddball task.** The average (bars with standard deviation) and individual values (dots) of the three groups for six different metrics are shown. P300 peak onset was defined as the first time point where a significant difference between targets and non-targets could be observed. It was set to 1000 ms if no such difference could be observed. Peak amplitude and latencies were determined in a 10-time bootstrapping with 80% of the data each time. Classification accuracies were determined in 5-fold chronological crossvalidation. Abbreviations: **n.s.** = not significant, **AUC** = area under the receiver operator curve.

After correcting for multiple testing, none of the changes reached significance anymore. Comparing the NACs with patients, we overall observed a decreased oddball performance for patients. It manifests itself in higher P300 peak latencies, a later P300 onset, lower P300 and N200 peak amplitudes and an inferior BCI classification accuracy. However, these quantities were not tested for significance to limit the number of statistical tests.

14 Patient-wise results for the communicative activity log (CAL)

In addition to the group-level analysis of the CAL which has been presented in the main paper, we show the patient-wise results in Figure 3.

As the partners of the patients assisted in the completion process, we required that the part of CAL which is normally completed by an external person, should not also be filled in by the partner, but instead by e. g. a speech therapist. Unfortunately, we did not receive a sufficient amount of these external ratings and thus, can only report on the self-rated part.

15 Laterality indices for different seed regions in the resting-state fMRI analysis

We calculated the laterality index for different seed regions to judge where the changes in functional connectivity (FC) mainly took place. Please find the formula to calculate the laterality index in the method section of the main paper. The laterality index ranges from -1 (only right-lateralized changes) to +1 (only left-lateralized changes). Table 3 shows the results. Observing that all seed regions have laterality indices above 0 shows that changes occurred mainly on the left hemisphere.

Supplementary Table 3: **Laterality indices for different seed regions.**

Seed position	Laterality index for voxels that showed increased FC	Laterality index for voxels that showed decreased FC	Laterality index for voxels that showed either increased or decreased FC
PCC	0.99	0.67	0.85
Precuneus	0.28	0.71	0.6
F3op	0.49	0.7	0.59
F3tri	0.98	0.61	0.83
F3orb	0.78	0.67	0.74
STGp	0.9	0.68	0.77

Abbreviations: **FC**: functional connectivity, **PCC**: posterior cingulate cortex, **F3op**: pars opercularis of the inferior frontal gyrus, **F3tri**: pars triangularis of inferior frontal gyrus, **F3orb**: pars orbitalis of the inferior frontal gyrus, **STGp**: posterior superior temporal gyrus.

References

Aschenbrenner S, Tucha O, Lange KW. Regensburg word fluency test. Göttingen: Hogrefe 2000;.

Baddeley A. Working memory. Science 1992;255(5044):556–559.

Kessels RP, Van Zandvoort MJ, Postma A, Kappelle LJ, De Haan EH. The Corsi block-tapping task: standardization and normative data. Applied neuropsychology 2000;7(4):252–258.

Lakens D. Calculating and reporting effect sizes to facilitate cumulative science: a practical primer for t-tests and ANOVAs. Frontiers in psychology 2013;4:863.

Lotte F, Larrue F, Mühl C. Flaws in current human training protocols for spontaneous brain-computer interfaces: lessons learned from instructional design. Frontiers in Human Neuroscience 2013 9;7(568):1–11.

Schreuder M, Höhne J, Blankertz B, Haufe S, Dickhaus T, Tangermann M. Optimizing event-related potential based brain–computer interfaces: A systematic evaluation of dynamic stopping methods. Journal of Neural Engineering 2013 5;10(3):036025.

Zimmermann P, Fimm B. A test battery for attentional performance. Applied neuropsychology of attention Theory, diagnosis and rehabilitation 2002;p. 110–151.
